# Non-Extensive Fragmentation of Natural Products and Pharmacophore-Based Virtual Screening as a Practical Approach to Identify Novel Promising Chemical Scaffolds

**DOI:** 10.3389/fchem.2021.700802

**Published:** 2021-08-02

**Authors:** Andrés Felipe Vásquez, Alejandro Reyes Muñoz, Jorge Duitama, Andrés González Barrios

**Affiliations:** ^1^ Grupo de Diseño de Productos y Procesos (GDPP), Department of Chemical Engineering, Universidad de Los Andes, Bogotá, Colombia; ^2^ Naturalius S.A.S, Bogotá, Colombia; ^3^ Grupo de Biología Computacional y Ecología Microbiana (BCEM), Department of Biological Sciences, Universidad de Los Andes, Bogotá, Colombia; ^4^ Max Planck Tandem Group in Computational Biology, Universidad de Los Andes, Bogotá, Colombia; ^5^ Systems and Computing Engineering Department, Universidad de Los Andes, Bogotá, Colombia

**Keywords:** fragment-based drug discovery, RECAP, virtual screening, pharmacophore modeling, property-based design, protein-ligand interactions, fragment library generation

## Abstract

Fragment-based drug design (FBDD) and pharmacophore modeling have proven to be efficient tools to discover novel drugs. However, these approaches may become limited if the collection of fragments is highly repetitive, poorly diverse, or excessively simple. In this article, combining pharmacophore modeling and a non-classical type of fragmentation (herein called non-extensive) to screen a natural product (NP) library may provide fragments predicted as potent, diverse, and developable. Initially, we applied retrosynthetic combinatorial analysis procedure (RECAP) rules in two versions, extensive and non-extensive, in order to deconstruct a virtual library of NPs formed by the databases Traditional Chinese Medicine (TCM), AfroDb (African Medicinal Plants database), NuBBE (Nuclei of Bioassays, Biosynthesis, and Ecophysiology of Natural Products), and UEFS (Universidade Estadual de Feira de Santana). We then developed a virtual screening (VS) using two groups of natural-product-derived fragments (extensive and non-extensive NPDFs) and two overlapping pharmacophore models for each of 20 different proteins of therapeutic interest. Molecular weight, lipophilicity, and molecular complexity were estimated and compared for both types of NPDFs (and their original NPs) before and after the VS proceedings. As a result, we found that non-extensive NPDFs exhibited a much higher number of chemical entities compared to extensive NPDFs (45,355 vs. 11,525 compounds), accounting for the larger part of the hits recovered and being far less repetitive than extensive NPDFs. The structural diversity of both types of NPDFs and the NPs was shown to diminish slightly after VS procedures. Finally, and most interestingly, the pharmacophore fit score of the non-extensive NPDFs proved to be not only higher, on average, than extensive NPDFs (56% of cases) but also higher than their original NPs (69% of cases) when all of them were also recognized as hits after the VS. The findings obtained in this study indicated that the proposed cascade approach was useful to enhance the probability of identifying innovative chemical scaffolds, which deserve further development to become drug-sized candidate compounds. We consider that the knowledge about the deconstruction degree required to produce NPDFs of interest represents a good starting point for eventual synthesis, characterization, and biological activity studies.

## Introduction

**FIGURE 1:**
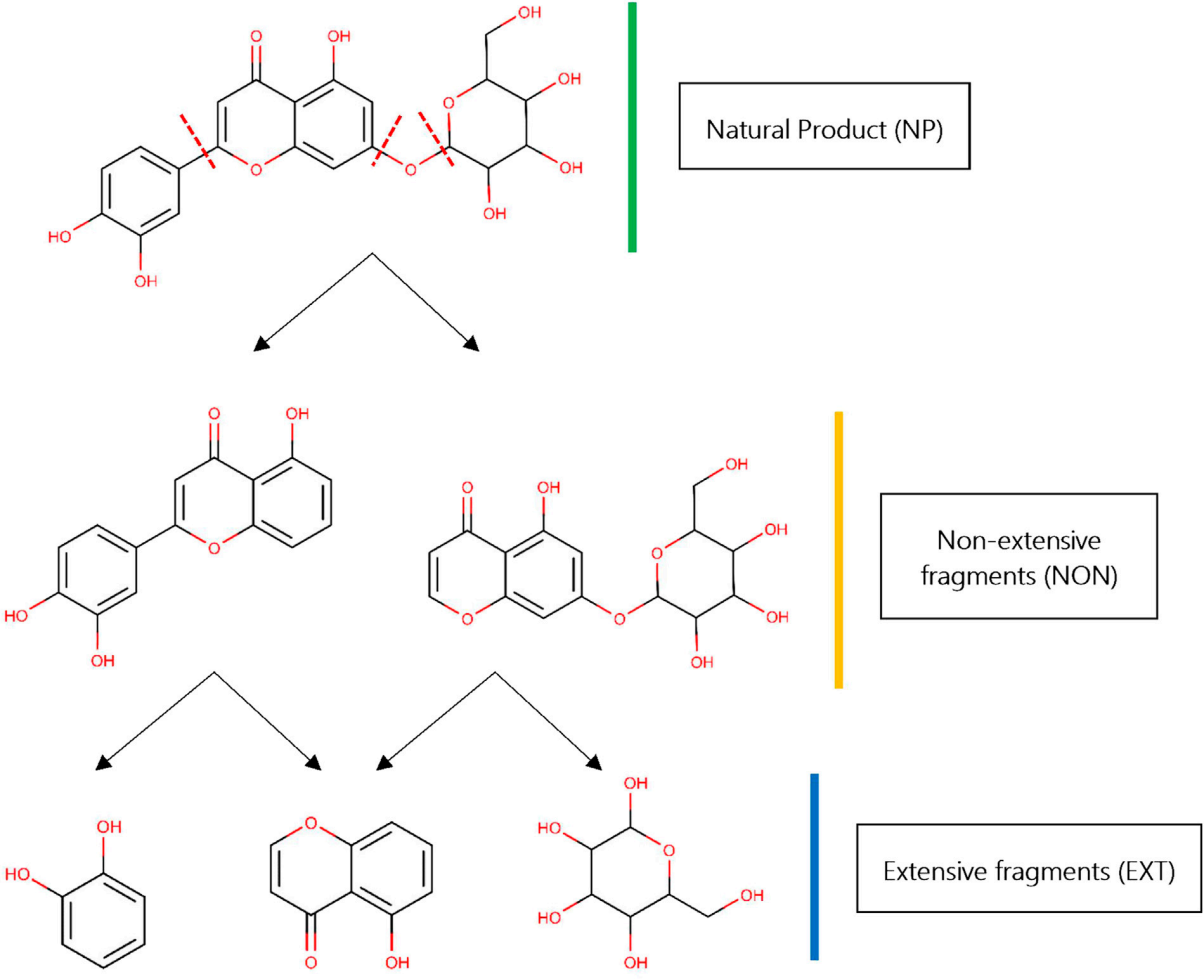
Fragmentation of NP libraries. Starting from a “parent” NP (colored green), two different kinds of fragments could be obtained: extensive fragments (colored gold) and “intermediate” or non-extensive fragments (colored blue). The NP shown in this example, *2-(3,4-dihydroxyphenyl)-5-hydroxy-7-{*[*3,4,5-trihydroxy-6-*(*hydroxymethyl*)*oxan-2-yl*]*oxy}-4H-chromen-4-one*, was taken from African medicinal plants database—AfroDB (Ntie-Kang et al., 2013). Red discontinuous lines depict the cleavage sites for RECAP in the original parent NP. All 2D chemical structures were visualized using the software MarvinView v15.1.19 provided by ChemAxon (https://www.chemaxon.com/).

Fragment-Based Drug Design (FBDD) is a well-established approach based on the screening of chemical fragments with low molecular weight (from 15 to 20 heavy atoms approximately), which are used to facilitate higher complementarity within a receptor binding site, more efficient exploration of the chemical space (Hall et al., 2015), and, ultimately, the identification of compounds able to bind efficiently to a particular biological target (Schulz and Hubbard, 2009; Erlanson et al., 2016). Since fragments may be produced by deconstructing chemical libraries, bond-cutting rules as the widely known RECAP have been used for this purpose (Lewell et al., 1998). RECAP rules theoretically handle the cleavage sites within a molecule in two different ways: a classical, extensive (i.e., exhaustive) that generates a collection of fragments as small as possible; and an alternative, non-extensive that generates all possible “intermediate” scaffolds by considering the cleavage sites in a systematic manner (Figure 1). Both types have been previously termed leaf and non-leaf nodes, respectively, by other authors (Fechner and Schneider, 2007).

Pharmacophore modeling is also a drug discovery approach increasingly employed for Virtual Screening (VS) campaigns (Chen et al., 2009). It consists of an ensemble of 3D stereo-electronic features shared by well-known active compounds and, hence, necessary to ensure a favorable interaction with a particular biological target of interest (Wallach, 2011; Sanders et al., 2012). Intriguingly, despite the increasing number of examples for pharmacophore modeling or FBDD reported in the last two decades, the implications of a combined implementation of these approaches have been poorly discussed and analyzed, except for a couple of research works published by (Teodoro and Muegge, 2011; Fechner and Schneider, 2007), respectively. However, we believe that several key aspects still remain unresolved. First, the extensive and non-extensive fragments have not been compared in terms of their chemical diversity, developability, or potency. Second, pharmacophore modeling, while employed as a screening tool of RECAP-derived fragments, has not been discussed as a possible cause of diversity reduction in the resulting chemical library. Finally, no NPs are listed in any of these studies as members of the chemical library under evaluation. The latter subject is critical because the often sought-after potential of scaffold hopping for both FBDD and pharmacophore modeling largely depends on the diversity of the screening library; thus, structurally complex compounds such as NPs are intended to increase this capability (Grabowski et al., 2008).

**FIGURE 2:**
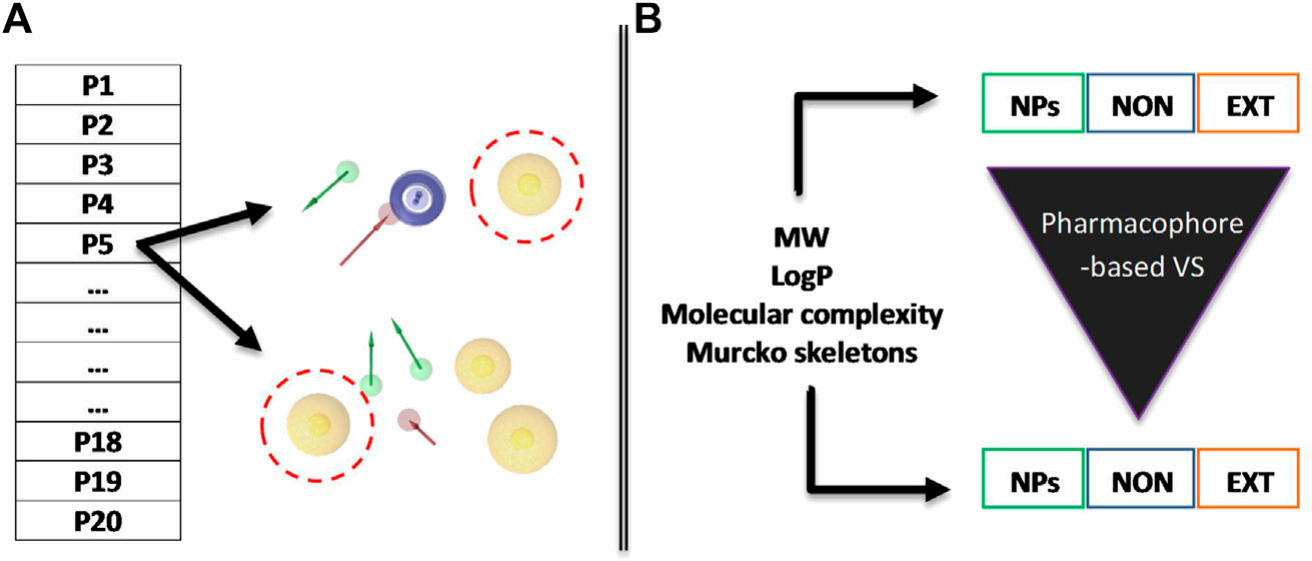
Schematic diagram of the methodological strategy used in this study. **(A)** Pairs of overlapping pharmacophore models were constructed and validated for each of the proteins of interest and then used for the VS procedures. **(B)** Before and after the VS, various parameters are estimated for the three different populations of chemical entities: original or parent NPs, non-extensive fragments (NON), and extensive fragments (EXT). The pharmacophore features are color-coded as described in the *Methodology* section depending on their inherent chemical nature. Red discontinuous spheres illustrate the overlapping features of the model included as an example.

In this research work, we developed a VS protocol combining RECAP-based FBDD and 3D pharmacophore modeling over a set of virtual libraries of NPs (Figure 2). First, we generated pairs of overlapping pharmacophore models for 20 different protein targets. Second, we evaluated several physicochemical features such as molecular weight (MW) and lipophilicity (logP) for 1) original natural products (NPs), 2) extensive natural-product-derived fragments (NPDFs), and 3) non-extensive NPDFs before and after VS using each pharmacophore model. The non-extensive NPDFs were shown to be more diverse, developable, and potent than extensive NPDFs, therefore, with a greater capacity to yield starting chemical moieties able to be developed into candidate compounds. We believe that these results will be highly useful in VS pipelines at early stages of the drug discovery and development process: they may accelerate not only the identification of innovative chemical scaffolds but also the prioritization of key decision-making criteria around the deconstruction of NPs (such as synthetic effort and patentability).

## Methodology

### Pharmacophore Models Generation

**FIGURE 3:**
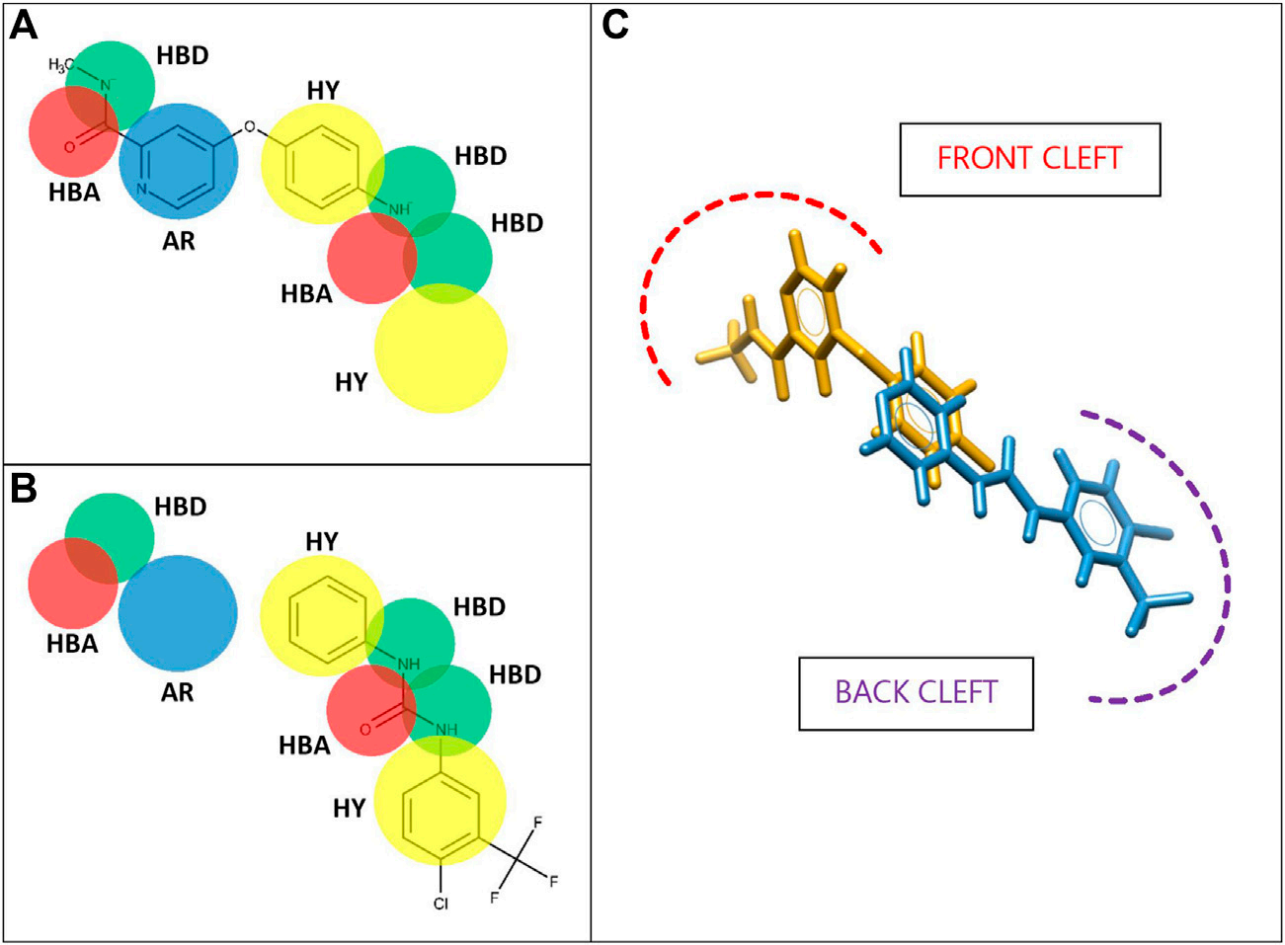
Proposed strategy for fragment-based VS procedures *via* overlapping pharmacophore models. Partial, segmented pharmacophore models are aimed at recognizing and capturing fragments interacting with the binding site of CDK2/VEGFR2 over close or overlapping zones, namely, front, and back cleft pockets **(A,B)**. Best scored fragments can be merged to eventually produce a drug-sized candidate compound **(C)** *.* HBA: H-bond acceptor; HBD: H-bond donor; HY: hydrophobic group; AR: Aromatic ring. All 2D chemical structures were visualized using the software MarvinView v15.1.19 (ChemAxon).

A set of ligand-based pharmacophore models was constructed and validated using the software Ligand Scout v4.4.1 (Wolber and Langer, 2005) for 20 protein targets of therapeutic interest. These targets were arbitrarily selected from the DEKOIS 2.0 virtual library (http://www.dekois.com/) as a representative sample of 1) the main protein classes and 2) the “challenging” dataset levels established by (Bauer et al., 2013) (Table 1). One additional pair of models recently described by our research group for CDK2/VEGFR2 was also included (Vásquez et al., 2020). We generated the models for each protein as overlapping pairs to promote an eventual development of NPDFs by a merging strategy (Hung et al., 2011; Scoffin and Slater, 2015) (Figure 3). Tolerance spheres for 3D pharmacophore features such as H-bond acceptor (HBA), H-bond donor (HBD), hydrophobic group (HY), aromatic ring (AR), positive ionizable, (PI), and/or negative ionizable (NI) features were considered in the construction of the models if they included the centers of chemical moieties of well-known active compounds with the corresponding chemical nature, as described in (Wolber and Langer, 2005). Therefore, each cluster of features was intended to represent the most relevant protein-ligand interactions. Pharmacophore fit score (also referred to as fitness score) was calculated in terms of how well the features in a particular model correctly match with the features of a given chemical compound and, hence, they were used to rank hit molecules retrieved from each model. The fit score was used to calculate the number of features successfully matched and the root-mean-square deviation (RMSD) between a pharmacophore model and the pharmacophoric points of the conformer for a query compound, which in turn is based on inter-feature distance sets (Euclidean function) compared in a pairwise manner (Markt et al., 2011). Additionally, we included exclusion volume spheres (XVols) in each model to represent areas already occupied by the equivalent protein receptor and, thus, inaccessible by the cognate ligand.

**TABLE 1:** Protein classes, short names, and full names of the protein targets included in this study.


Protein class	Short name	Full name
Proteases	ACE	Angiotensin-I-converting enzyme
	CATL	Cathepsin L
	CTSK	Cathepsin K
Hydrolases	ACHE	Acetylcholinesterase
	HSP90*	Heat shock protein 90
	KIF11	Kinesin family member 11
Kinases	AURKA**	Aurora A kinase
	CDK2**/VEGFR2**	Cyclin-dependent kinase 2/vascular endothelial growth factor receptor 2
	EGFR**	Epidermal growth factor receptor
	IGF1R**	Insulin-like growth factor 1 receptor
	MK2	MAPK-activated protein kinase 2
	PIM-2	Serine/threonine-protein kinase PIM-2
	PRKCQ	Protein kinase C theta
	ROCK-1	Rho-associated protein kinase-1
Transferases	QPCT	Glutaminyl-peptide cyclotransferase
Ligases	MDM2	p53-binding protein MDM2
Oxidoreductases	COX2	Cyclooxygenase-2
	DHFR**	Dihydrofolate reductase
	HMGR	HMG-CoA reductase
	INHA**	Enoyl ACP Reductase

The complete set of active and inactive (decoy) compounds for each protein was directly downloaded from DEKOIS 2.0 virtual library (http://www.dekois.com/), maintaining the corresponding names. The set of proteins are listed according to their enzyme commission numbers (EC) numbers. Moderately challenging (*) and highly challenging (**) targets were included according to the reported by (Bauer et al., 2013) in the construction of the benchmark set DEKOIS 2.0.

For the training of the pharmacophore models, we used a structurally diverse benchmark set of active and inactive (decoys; 30 per active) compounds from the chemical library DEKOIS 2.0 (Bauer et al., 2013). Specifically, ligands in this database were selected, processed, and curated to avoid substructures frequently causing false positives, chemically reactive behaviors, negligible bioactivities as actives, and binding to different sites of the same target, among other known biases. The decoys, in contrast, were selected based on an approach referred to as the LADS filter intended to avoid latent actives in the decoy set and, therefore, any artificial enrichment bias (Bauer et al., 2013). Each model encompassed three or more features (tolerance spheres). We generated an ensemble of 100 conformers for each active and decoy compound, which were retrieved if, as a minimum, one conformer successfully satisfied the corresponding pharmacophore model. Next, the pharmacophore features were checked by visual inspection and iteratively manually refined if required (e.g., deletion/addition and increase/decrease of weight or tolerance sphere radius) as discussed in (Laggner et al., 2009; Schaller et al., 2020). Receiver Operating Characteristic (ROC) curves were then constructed by plotting the fraction of true positives out of the total actual positives (i.e., the True Positive Rate or TPR) vs. the fraction of false positives out of the total actual negatives as described in (Rizzi and Fioni, 2008). Finally, to validate each model, we calculated the Area Under Curve (AUC) from each ROC curve at the first (top) 1, 5, 10, and 100% of the database, which was composed of corresponding actives and decoys ranked in terms of their pharmacophore fit scores (Markt et al., 2011).

### Virtual Deconstruction of Natural Product Libraries

A virtual chemical library was produced using four different NP virtual databases, namely, the databases TCM (http://zinc12.docking.org/pbcs/tcmnp), NuBBE (http://zinc12.docking.org/pbcs/nubbenp), UEFS (http://zinc12.docking.org/pbcs/uefsnp), and AfroDb (http://zinc12.docking.org/pbcs/afronp), which can be accessed from the freely available ZINC database (Irwin and Shoichet, 2005; Irwin et al., 2012). These 41,472 unique compounds were fragmented into NPDFs using an original and an *in-house* modified version of the script Fragmenter v1.0 (Gruening and Patel, 2012), respectively (Supplementary Data 1), both implementing the RECAP method originally proposed by (Lewell et al., 1998). While the first version only generated extensive NPDFs, the second one allowed obtaining every possible combination of both extensive and non-extensive NPDFs. Regardless of the script used for fragmentation, all retrieved NPDFs were tracked to their corresponding NP parent molecule.

### Pharmacophore-Based Screening of Natural Product-Derived Fragments

A VS protocol was developed with the original NPs and the NPDFs using Ligand Scout v4.4.1 (Wolber and Langer, 2005). We generated 100 conformers for each chemical entity and employed the screening mode “stop after the first matching conformation” to identify hits successfully satisfying the corresponding pharmacophore model as stated in (Goldmann et al., 2015; Debnath et al., 2016, 2019). The pharmacophore fit score was then retrieved for each hit compound. Also, Mona v2.1.3 from BioSolveIT (Hilbig et al., 2013; Hilbig and Rarey, 2015) was used as an interactive tool to estimate the molecular weight (MW) and lipophilicity (logP) of the resulting small-molecule datasets. Duplicates were removed before and after the screening by considering instances with the same topology as equal; however, those with different stereoisomeric, tautomeric, and protomeric forms were considered as independent chemical entities. We used DataWarrior v5.0.0 (Sander et al., 2015) to estimate the molecular complexity and the number of Murcko (also called Bemis-Murcko) skeletons of the datasets. Murcko skeletons are a generalized form of Murcko scaffold, in which a molecular framework contains all plain ring systems of the query molecule and their corresponding connections, removing substituents and replacing hetero atoms with carbon atoms (https://openmolecules.org/). MarvinView v19.27.0 from ChemAxon was used to convert SDF files to CSV files for further analysis. All of these parameters were compared for the original NPs and both types of NPDFs before and after the VS proceedings.

## Results

In the first part of our study, we constructed and statistically validated 20 pairs of overlapping pharmacophore models (one per each protein). According to the ROC curve analysis, the initial models for these target proteins (Supplementary Data 2) showed AUC values between 0.72 and 0.98, respectively, for 100% of the database composed of active and decoy compounds (Table 2). The AUC in earlier steps, especially at 1% of the database, was equal to or close to 1.0 for most of the pharmacophore models. Likewise, the TPR for each model was also close to 1.0 for all cases. Finally, each pharmacophore model was divided into two different overlapping models (Supplementary Figure 1) and employed during the subsequent VS procedures.

**TABLE 2:** Summary of performances of pharmacophore hypotheses generated for the VS protocol.


Protein	AUC				TPR
	1%	5%	10%	100%	
**ACE**	1.00	1.00	1.00	0.96	0.98
**ACHE**	0.41	0.88	0.94	0.87	0.85
**AURKA**	0.92	0.97	0.95	0.80	0.88
**CATL**	1.00	1.00	1.00	0.90	0.88
**CDK2/VEGFR2**	1.00/0.94	0.98/0.99	0.98/0.99	0.87/0.84	1.00/0.98
**COX2**	1.00	1.00	1.00	0.91	0.88
**CTSK**	1.00	1.00	1.00	0.92	0.90
**DHFR**	0.00	0.45	0.70	0.83	0.98
**EGFR**	1.00	1.00	1.00	0.84	0.68
**HMGR**	1.00	1.00	1.00	0.98	1.00
**HSP90**	1.00	1.00	1.00	0.88	0.90
**IGF1R**	0.25	0.85	0.89	0.72	0.78
**INHA**	0.67	0.80	0.84	0.78	0.83
**KIF11**	0.83	0.94	0.94	0.85	0.83
**MDM2**	1.00	1.00	1.00	0.85	0.98
**MK2**	1.00	1.00	1.00	0.90	0.98
**PIM-2**	0.92	0.98	0.96	0.80	0.65
**PRKCQ**	1.00	1.00	1.00	0.83	0.85
**QPCT**	0.92	0.92	0.94	0.84	0.83
**ROCK**	1.00	1.00	0.99	0.76	0.70

AUC=Area Under ROC Curve; TPR = True Positive Rate.

Once the virtual chemical libraries were deconstructed, we obtained a non-repetitive set of 11,525 extensive and 45,355 non-extensive NPDFs. Along with the set of original NPs, a total of almost 10^5^ chemical entities was reached for the analysis pre- and post-screening, which represents an increase of more than 16% in the number of chemical entities for the non-extensive NPDFs compared to the original NPs library (Supplementary Data 3). On the other hand, 9,755 NPs could not be deconstructed (at least once) using RECAP rules, accounting for 23% of our entire virtual chemical library. All of the resulting compound sets (i.e., extensive fragments, non-extensive fragments, and original NPs) were subsequently taken to the VS using their corresponding pair of pharmacophore hypotheses (models) per protein (Table 3). For each protein included in the VS, the number of screened non-extensive NPDFs was higher than that of extensive NPDFs and original NPs (Supplementary Data 4).

**TABLE 3:** Analysis results of the number of non-repetitive hits obtained from each compound set for the 20 pairs of overlapping pharmacophore models (arbitrarily tagged here as left and right).

Protein	Left pharmacophore model				Right pharmacophore model				Left U Right		
	EXT	NON	NP	Total	EXT	NON	NP	Total	EXT	NON	NP
**ACE**	7,183	35,314	27,974	70,471	981	4,334	4,503	9,818	7,346	35,566	28,414
**ACHE**	213	2,498	1,128	3,839	2,800	14,793	9,751	27,344	2,879	15,844	10,213
**AURKA**	2,329	13,784	5,414	21,527	1,377	6,009	10,277	17,663	2,956	16,489	12,525
**CATL**	6,436	31,317	17,717	55,470	4,463	24,639	24,509	53,611	7,667	37,165	28,653
**CDK2/VEGFR2**	210	860	643	1713	17	211	125	353	225	1,053	757
**COX2**	2,095	13,143	9,144	24,382	1,476	7,708	5,206	14,390	2,963	16,978	11,918
**CTSK**	2,311	13,238	9,208	24,757	3,156	18,148	14,831	36,135	4,721	25,405	19,767
**DHFR**	1,058	11,475	7,482	20,015	1,983	11,805	8,940	22,728	2,219	16,194	11,303
**EGFR**	18	108	94	220	910	5,287	3,638	9,835	926	5,372	3,716
**HMGR**	7,147	26,979	22,701	56,827	1,325	4,603	3,998	9,926	7,329	27,653	23,255
**HSP90**	2,886	18,047	13,676	34,609	2,941	18,377	14,009	35,327	3,365	20,451	15,335
**IGF1R**	2,971	21,080	14,096	38,147	7,551	32,839	27,092	67,482	8,131	36,889	29,594
**INHA**	700	6,554	4,414	11,668	1,306	7,342	4,996	13,644	1,709	11,873	8,020
**KIF11**	5,179	21,183	16,952	43,314	577	4,273	2,389	7,239	5,598	23,784	18,439
**MDM2**	6,555	30,256	25,463	62,274	6,099	28,970	23,974	59,043	7,018	31,805	27,199
**MK2**	5,476	28,419	20,649	54,544	6,048	28,226	23,064	57,338	7,851	37,280	28,750
**PIM-2**	87	528	466	1,081	1,061	4,835	3,723	9,619	1,127	5,210	4,112
**PRKCQ**	6,700	25,980	26,739	59,419	6,932	32,773	23,026	62,731	8,855	36,665	31,523
**QPCT**	1,770	10,346	7,016	19,132	1,018	7,667	5,081	13,766	2,349	14,982	10,106
**ROCK-1**	130	1,101	839	2,070	366	3,056	2,244	5,666	434	3,638	2,699

EXT = extensive fragments; NON = non-extensive fragments; NP = original natural products.

**FIGURE 4:**
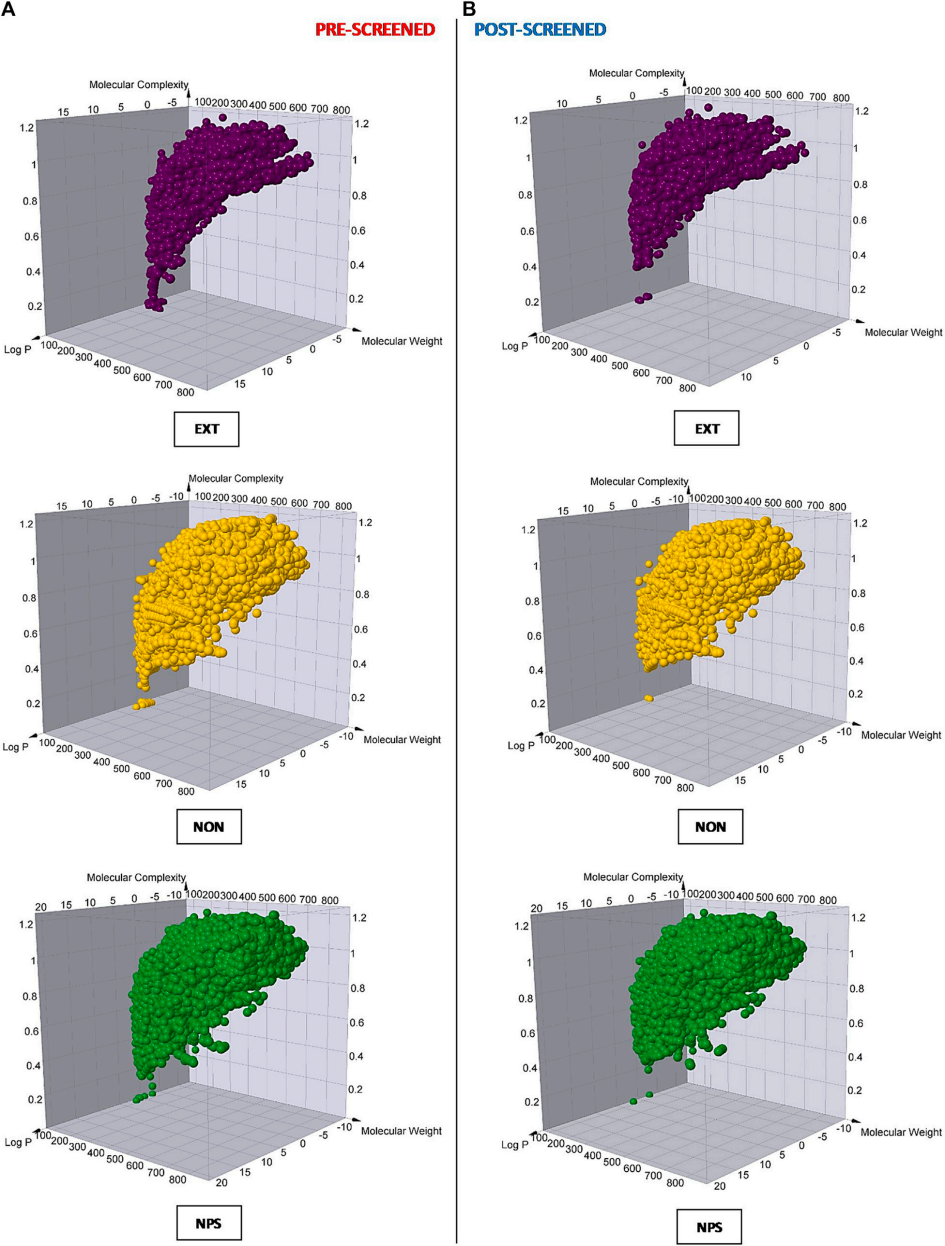
Molecular diversity analysis for each compound set of study. The molecular complexity, logP, and molecular weight were calculated for each chemical entity (illustrated as purple, gold, and dark-green spheres, respectively) in the three different sets of compounds, which were analyzed before **(A)** and after **(B)** the VS protocol. EXT = extensive fragments; NON = non-extensive fragments; NP = original natural products.

To continue evaluating the different groups of resulting hits, we developed a comparative analysis of Murcko skeletons, molecular weight, lipophilicity, and molecular complexity before and after the VS protocol for each of them. As a result, we observed that the non-extensive NPDFs occupied a similar zone of an estimated 3D chemical space compared to original NPs, but a much greater zone compared to extensive NPDFs in each case (Figure 4), which was also consistent with other physicochemical features (Supplementary Table 1). In line with these results, we observed a remarkably lower repetitive pattern in the non-extensive NPDFs than in the extensive NPDFs based on the analysis of the number of duplicates (Supplementary Table 2). Although the number of Murcko skeletons of the non-extensive NPDFs was, as expected, lower than that of the original NPs (about 30%), it doubled those of extensive NPDFs (Table 4). No large differences were observed for the number of Murcko skeletons before and after the VS procedures. However, even though the range of all these physicochemical features was narrower for non-extensive NPDFs than that for the original NPs, as expected, it was wider than that for the extensive NPDFs.

**TABLE 4:** Number of Murcko skeletons for each compound set before and after the VS protocol.

Compound database	Murcko skeletons	
	Before VS	After VS
EXT	2,909	2,869
NON	6,086	6,053
NP	9,285	9,153

EXT = extensive fragments; NON = non-extensive fragments; NP = original natural products.

**FIGURE 5:**
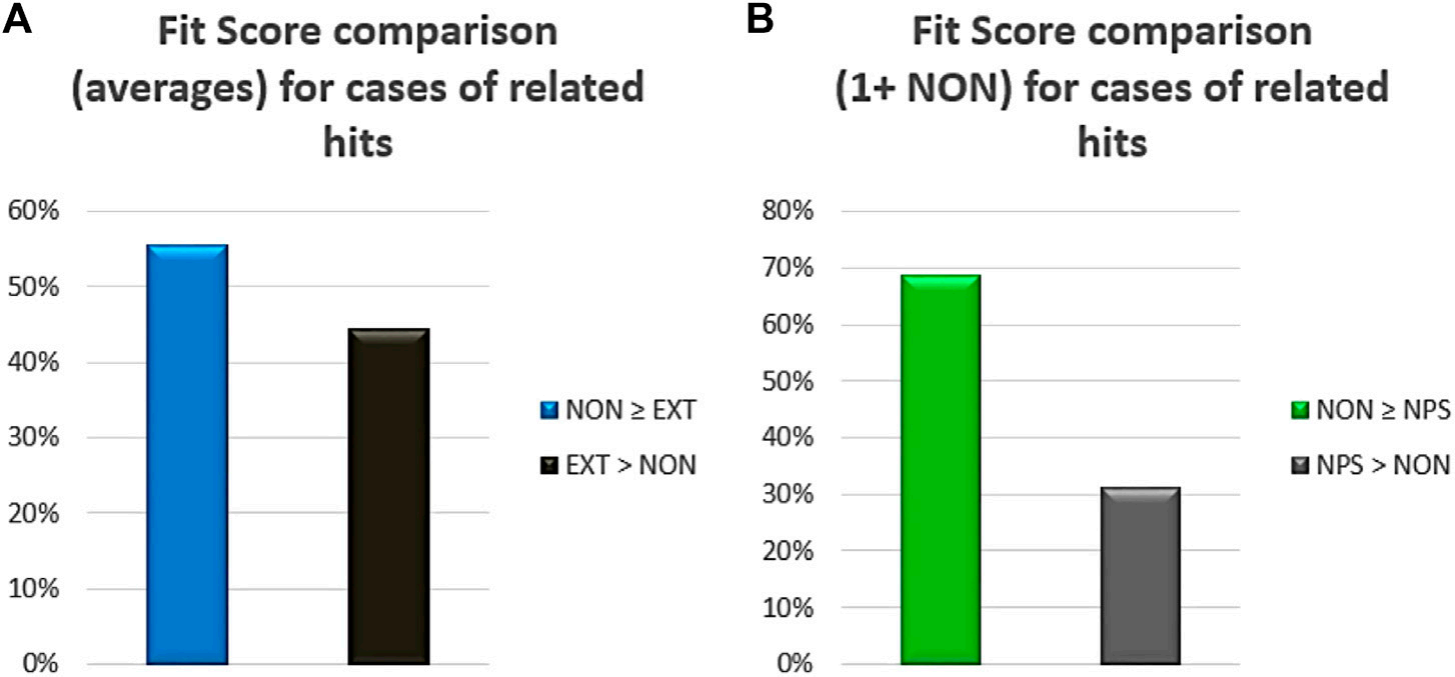
Fit score comparison between non-extensive (NON) and either extensive NPDFs (EXT) or original NP compounds (NPs). Percentages in the first case **(A)** were calculated based on the comparison of averages just for the hits related to each other (i.e., sharing the same original NP). In the second case **(B)**, percentages were calculated based on the number of cases with one or more non-extensive NPDFs being greater or lower than their corresponding NP (also recognized as a hit).

Finally, a comparative framework of the potency was established using the pharmacophore fit score of the chemical entities recognized as hits after the VS protocol. If extensive and non-extensive NPDFs hits were related to each other (i.e., sharing the same original NP), the comparison between their corresponding averages indicated that the non-extensive NPDFs had better fit scores in 56% of the cases (Figure 5A). Interestingly, when both non-extensive NPDFs and their respective parental NPs were recognized as hits, at least one non-extensive NPDF (i.e., one or more) exhibited a higher fit score almost 70% of the time (Figure 5B). A pair of chemical moieties (non-extensive NPDFs) retrieved from the VS protocol is illustrated in Supplementary Figure 2, which includes 2-hydroxy-5-(3-methylbut-2-en-1-yl)-4-(propan-2-yl) cyclohepta-2,4,6-trien-1-one (ZINC5458295) and (2S)-2-amino-4-methyl-N-(naphthalen-2-yl) pentanamide (ZINC57384) (https://zinc.docking.org/substances/).

## Discussion

### Pharmacophore Models Were Able to Distinguish Between Active and Decoy Compounds

A set of 20 pairs of overlapping pharmacophore models was constructed and validated for the proteins of study. This step pretends to pose a helpful path to favor not only the detection of potential NPDFs as potential hits in the VS procedures but also their eventual merging. In this specific process, there is a greater probability that fragments maintain the same conformation compared to the final merged compound (which is critical to estimate pose and binding energy) (Schade, 2007), and that the design of a linker or the sequential addition of NPDFs to occupy the pockets of the target protein is not required (Lanz and Ried, 2015). While merging any NPDFs for each protein is certainly the next natural step to identify virtual candidates, we consider that this task is beyond the scope of this particular study and, therefore, it has not been included. However, the successful merging of one protein system reported here (CDK2/VEGFR2) has been proved in a previous study focused on the design of dual inhibitors (Vásquez et al., 2020). In this previous study, a set of NPDFs, each one representing overlapping zones in both enzymes, was combined in drug-sized compounds able to occupy the active site of these highly challenging targets. The entire merging strategy allowed counting on middle-sized fragments occupying more than one pocket in each enzyme (most probably using non-extensive fragments), which not only improved the chance of having a more similar positioning before and after the combining procedure but also proved appropriate for increasing chemical diversity in the section covering the overlapping region (interacting with a critical gatekeeper (GK) residue) (Vásquez et al., 2020).

On the other hand, and according to ROC curve analysis, the high AUC values obtained for the pharmacophore models of this study (especially in early “top” stages) demonstrated that the employed set of models was suitable to discriminate between active and decoy compounds and, hence, could be employed for the VS protocols. The strategical advantage of obtaining high early AUC values has been identified by other authors as critical in VS campaigns (Venkatraman et al., 2010; Scior et al., 2012). It is worth mentioning that we decided to primarily use enzymes as protein targets because they are widely known to have a well-defined and preformed concave binding cavity (active site) (Kahraman and Thornton, 2008), which are opposed to the protein-protein interfaces (PPIs) that usually have a shallow, flat, spacious, highly hydrophobic, and rather poorly structured binding site (Laskowski et al., 1996; Bauer et al., 2013). In the same line of thought, enzyme active sites tend to bind to ligands with physicochemical features similar to those of well-known drugs or drug-like compounds in which, for example, H-acceptors and H-donors account for two-thirds and a third of H-bonds in a typical protein-ligand complex (Kahraman and Thornton, 2008), This is in contrast to what is normally observed for PPIs, which tend to bind to ligands with a very high molecular weight and lipophilicity (Higueruelo et al., 2012; Bauer et al., 2013). Thus, we strongly believe these selection criteria for the datasets facilitated the analysis, which otherwise would have been highly complex and uneven.

### Non-Extensive Natural-Product-Derived Fragments Played a Major Role in the Database of Study Before and After Virtual Screening

From the NPs deconstruction protocol, two different facts could be elucidated. On the one hand, the remarkable generation of NPDFs, -especially non-extensive-, suggests a key role of these in increasing the number of chemical entities during the NP deconstruction and, hence, the entire virtual database eventually used during the VS step. Whereas increasing the library size is certainly favorable for a VS, it is even more advantageous in the context of FBDD because a much lower number of fragment-sized compounds is required to explore the theoretical chemical space compared to drug-like compounds (∼10^7^ vs. ∼10^60^, respectively) (Bohacek et al., 1996; Fink et al., 2005; Bembenek et al., 2009). (Over et al., 2012; Harvey et al., 2015). However, it is relevant to notice that the relatively low number of unique, extensive NPDFs here obtained is due, in part, to the fact that 1) an important part of the evaluated NPs is not responsive to RECAP-based fragmentation and 2) extensive NPDFs are relatively simple structures and, therefore, tend to be much more repetitive and uninformative. This last fact was demonstrated by the higher number of duplicates in extensive NPDFs compared to non-extensive NPDFs. On the other hand, we believe that the concentration of extensive NPDFs within a remarkably limited range of molecular complexity and Murcko skeletons might be related to the degree of responsiveness to RECAP rules. Therefore, those NPs not being deconstructed by RECAP could represent either a potentially limited synthetic accessibility or a limitation of the technique once applied in certain types of NPs. Future evaluation of NP databases submitted to other deconstruction strategies will be pertinent to understand their differences from standard drug-like databases.

After VS procedures, our results showed that the number of non-extensive NPDFs selected as hits was higher than that of NPs and extensive NPDFs. While it is tempting to speculate that this result was expected because the number of non-extensive NPDFs was higher than that of the other two groups of chemical entities (even before the VS), our results demonstrated that their fit scores have the same driving trend. Hence, since these scores were also greater than for most of the extensive NPDF hits sharing the same original NP and, in many cases, their original NP (also selected as a hit), it is evident that non-extensive NPDFs have a selective advantage for the models employed in the VS. Interestingly, even if it is intuitive to expect that non-extensive NPDFs could match more features of each model than extensive NPDFs, this hypothesis does not explain why they could exhibit better fit scores than their structurally related NPs. In line with this observation, our results suggest that an appropriate spatial conformation of the features is also critical to avoid problematic steric clashes in the model, including X-vol volumes describing the limits of the target receptor surface, as described in (Toba et al., 2006; Al-Nadaf and Taha, 2011). Consequently, a trade-off between the minimum features to be selected and the configuration required to match them in a 3D space appears to play a key role behind the advantages of the non-extensive fragmentation of NPs.

### Pharmacophore-Based Virtual Screening Could Maintain the Molecular Diversity of Compounds

The pharmacophore modeling protocol applied in this research played a key role during the VS procedures. Our results have shown that the pharmacophore-based strategy was able to maintain, even for low-to-medium-sized NPDFs, an effective trade-off between universality and specificity without jeopardizing the diversity of the hit library, as shown in other studies (Langer and Wolber, 2006; Laggner et al., 2009). This is especially critical for pharmacophore models modeling methods in which chemical feature constraints rely on chemical functionalities (mainly supporting universality) instead of molecular graph descriptors, (primarily supporting specificity) (Supplementary Table 3) (Wolber and Langer, 2005; Langer and Wolber, 2006). The retrieval of molecules structurally different to well-known active compounds-and to each other-has previously been discussed as a crucial objective to be achieved in a VS (Grabowski et al., 2008; Stocks et al., 2009; Markt et al., 2011), especially because obtaining different starting scaffolds supposes a larger developability advantage for the identified hit compounds (Scior et al., 2012). However, it might be possible that many chemical entities closely structurally related to their original NP are included within the set of non-extensive fragments and, consequently, partially facilitate the screening of NPDFs that are not necessarily small and poorly structurally diverse.

### Non-Extensive Natural-Product-Derived Fragments Proved to Have Better Potency Profiles Than Extensive Natural-Product-Derived Fragments and Natural Products

From the fit score evaluation for the three different compound sets, we discriminated two different elements. On the one hand, the non-extensive NPDFs hits proved to be more potent, in average, than the extensive NPDFs hits sharing the same NP, which was expected because of their higher molecular weight and, thus, the higher number of non-hydrogen atoms able to match the pharmacophore features (tolerance spheres). On the other hand, however, the fact that one or more non-extensive NPDF hits were more potent than their relative NP hits suggests that even if they share chemical moieties required to match the pharmacophore features, many resulting NPs hits are more limited in their pose. This result also implies the presence of flexibility constraints to satisfy the pharmacophore features and, possibly or alternatively, the steric effects of the binding pocket (recognized by interacting with exclusion volume spheres). In line with this observation, the existence of significant steric protein-ligand clashes has certainly been considered in previous reports as a critical part of pharmacophore refinement to improve selectivity (Toba et al., 2006; Leeson and Springthorpe, 2007; Metzler et al., 2008).

### Non-Extensive Fragments Could Have a Large Potential in the Context of Rule of Three and Ligand Efficiency Metrics

Under a developability criterion, non-extensive fragments were observed to adjust well to several features established by Astex’s Rule of Three (Ro3), except by two noticeable violations related to the molecular weight (MW) and H-acceptors (HBA). Interestingly, a remarkable increase in these two properties has been reported from the beginning of the 1900s for drugs developed from a natural prototype: it is especially certain for anti-infectious and anti-cancer agents whose major route of administration is not oral but injectable (Camp et al., 2015). A number of recently published research reports have discussed that NPs are compounds usually going beyond the rule of five (bRo5) in various features (primarily MW), which, by extension, affects the criteria considered by the Ro3 (Köster et al., 2011; Degoey et al., 2018; Tyagi et al., 2020). However, we should take also into account the importance of evaluating fragments (during their development to a bigger final candidate compound)as part of a whole molecule and not uniquely on an individual basis during their development to a bigger final candidate compound. This is particularly true for decision-making criteria such as ligand efficiency (LE) metrics, which have typically been guided by an overemphasis in potency (Hopkins et al., 2014). Within this frame of reference, novel approximations of LE metrics such as the RGC model proposed recently for our research group show us that even fragments exhibiting a low-to-moderate LE might be “rescued”(and hence become part of a drug-sized candidate) if other partner high-LE fragments are considered (Vásquez and González Barrios, 2019). We believe that it is especially true for non-extensive NPDFs because of their chemical diversity and potency compared to extensive NPDFs. However, we are not unaware that both types of fragments might play a complementary role as a source of novel chemical scaffolds depending on how much a NP needs to be deconstructed, as explained in the following section.

### Retrosynthetic Combinatorial Analysis Procedure-Based Fragmentation and Pharmacophore-based Virtual Screening Might Be Strategical to Guide the Synthesis of Natural-Product-Derived Fragments

**FIGURE 6:**
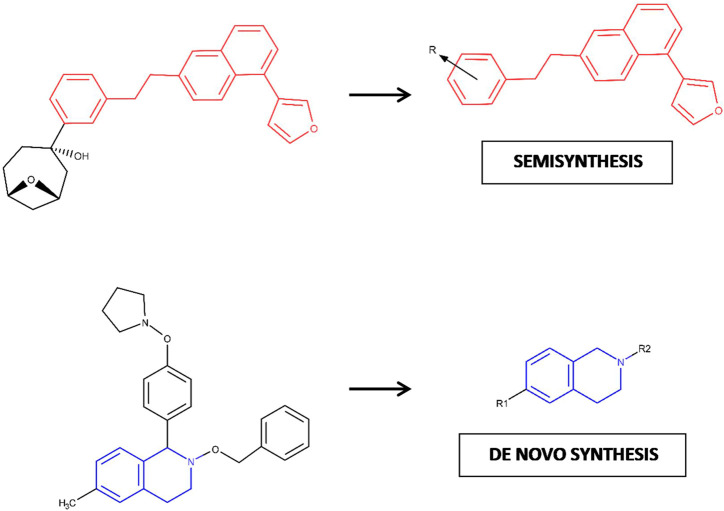
Hypothetic model proposed for the decision-making process in chemical synthesis based on the degree of required deconstruction (fragmentation) for one NP compound. The fragments of interest in each case are depicted here as chemical 2D substructures colored red and blue, respectively.

We believe that an additional implication of the combined approach applied in this study involves the possibility of evaluating if a particular NP should be deconstructed (and estimating how much) to generate a chemical entity able to satisfy a pharmacophore model and become part of a drug-sized compound. As shown in Figure 6, depending on whether or not a NPDF hit is identified as a potential substructure of a parent NP (and its corresponding synthetic effort degree), the best route to follow might involve either semisynthesis or *de novo* synthesis (Nising and Bräse, 2008; Haustedt and Siems, 2016; Borra et al., 2018). This fact is in line with the possibility of “scavenging” as much as possible of the chemical structure of a NP, which supposes an advantage because NPs usually exhibit high levels of complexity (Chen et al., 2019). Interestingly, since the benefits offered by FBDD strongly depend on the diversity of the chemical library to be used (Morrison et al., 2020), the results obtained in this study contribute to emphasizing the potential synergy of combining FBDD and NPs: while the diversity of the chemical library of interest is harnessed, its structural complexity is reduced until a synthetically accessible substructure can be found.

## Conclusion

In the present work, we have investigated the potential of combining the non-extensive RECAP deconstruction of NPs and pharmacophore modeling to identify novel, diverse, and potent fragments with promising chemical scaffolds. This is a rapid, intuitive, and effective strategy that could be implemented in future VS campaigns, considering the capacity of the non-extensive fragmentation to provide scaffolds of interest (compared to the classical extensive fragmentation) and the role of the 3D pharmacophores to favor scaffold hopping while maintaining the chemical diversity of the original NPs. Likewise, our results reveal that the entire cascade protocol is helpful to understand the deconstruction degree associated with a particular NP (i.e., the necessary steps to generate a fragment of interest), which we consider as highly advantageous to guide synthetic follow-up studies and, hence, conduct the development of candidate NPDFs into drug-sized compounds.

## Data Availability

The datasets presented in this study can be found in online repositories. The names of the repository/repositories and accession number(s) can be found below: https://doi.org/10.6084/m9.figshare.14794863.

## Abbreviations

AfroDb, African Medicinal Plants database; AR, aromatic ring; AUC, area under curve; CSV, comma-separated values; DEKOIS, Demanding Evaluation Kits for Objective In Silico Screening; EXT, extensive fragments; FBDD, fragment-based drug design; HBA, H-bond acceptor; HBD, H-bond donor; HY, hydrophobic group; LE, ligand efficiency; logP, octanol-water partition coefficient; MW, molecular weight; NI, negative ionizable; NON, non-extensive fragments; NP, natural products; NPDF, natural product-derived fragment; NuBBE, Nuclei of Bioassays, Biosynthesis, and Ecophysiology of Natural Products; PI, positive ionizable; PKI, protein kinase Inhibitor; RECAP, retrosynthetic combinatorial analysis procedure; RMSD, root-mean-square deviation; ROC, Receiver Operating Characteristic; SDF, structure data file; TCM, Traditional Chinese Medicine; TPR, true positive rate; UEFS, Universidade Estadual de Feira de Santana; VS, virtual screening.
