# Supplementary material for: Non-Extensive Fragmentation of Natural Products and Pharmacophore-Based Virtual Screening as a Practical Approach to Identify Novel Promising Chemical Scaffolds
Source: Front Chem. 2021 Aug 2;9:700802. doi: 10.3389/fchem.2021.700802 (PMC8377161; doi:10.3389/fchem.2021.700802)
Supplement: Supplementary file 1 [file DataSheet1.docx]

Supplementary Material

| **Protein** | **Left Pharmacophore model** | **Right Pharmacophore model** |
| --- | --- | --- |
| **ACE** | 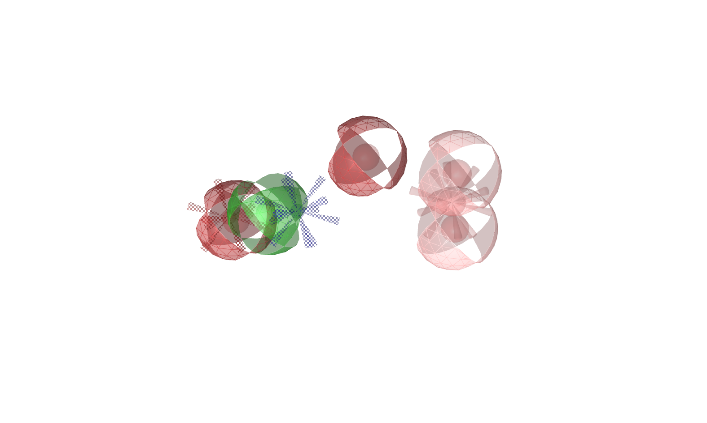 | 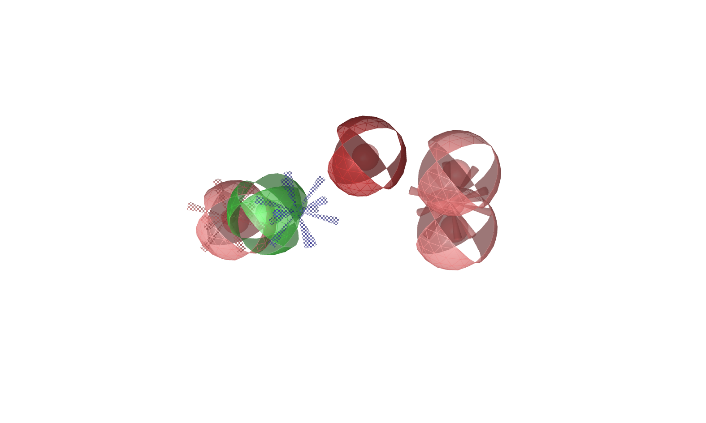 |
| **ACHE** | 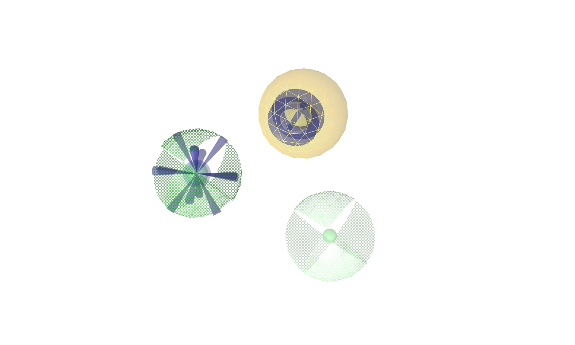 | 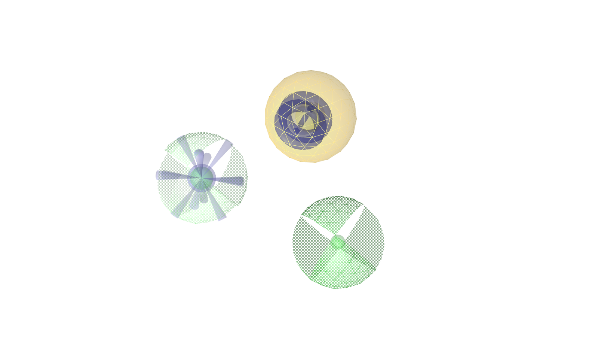 |
| **AURKA** | **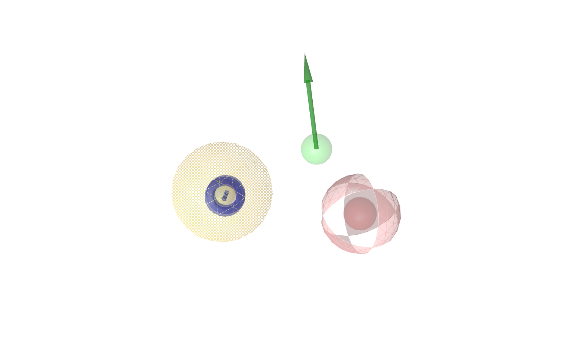** | **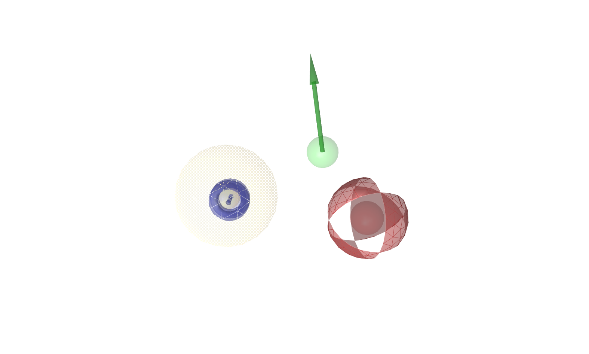** |
| **CATL** | **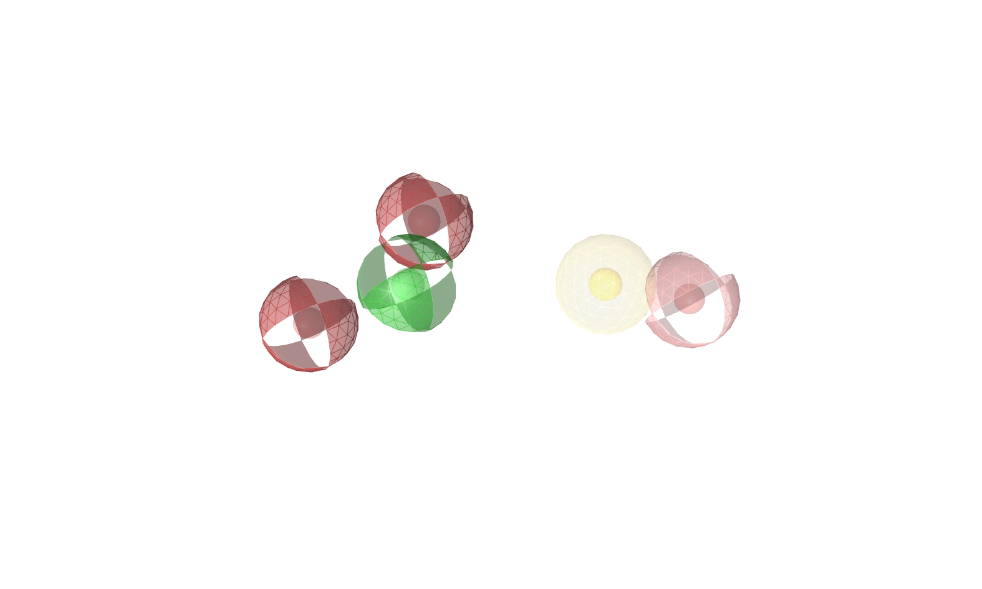** | **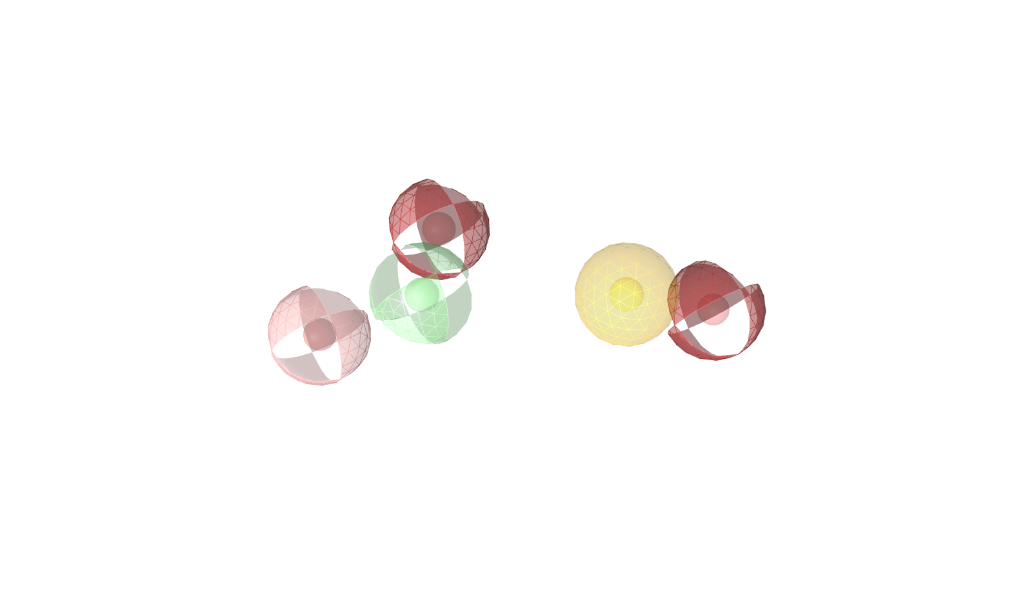** |
| **CDK2/VEGFR2** | **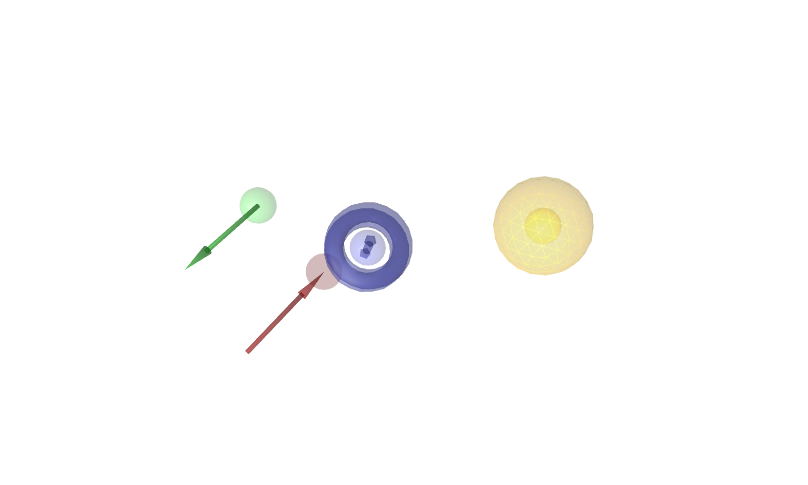** | **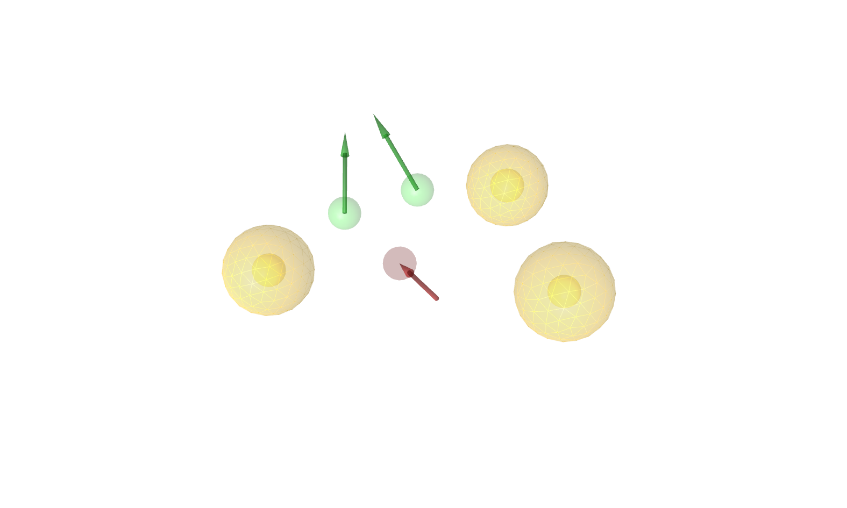** |
| **COX2** | **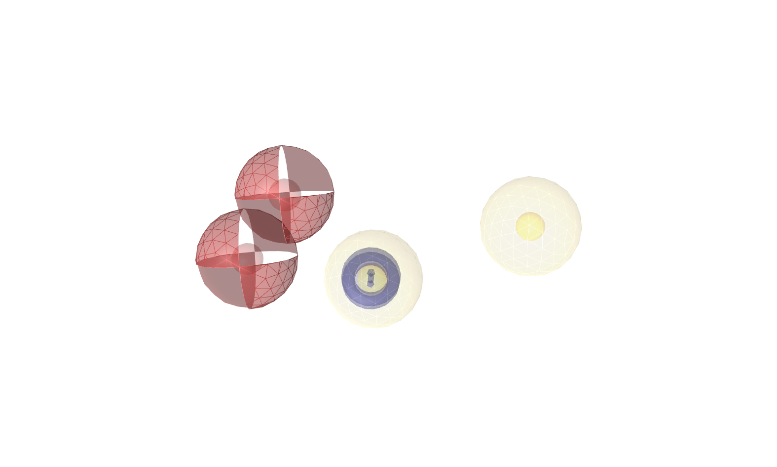** | **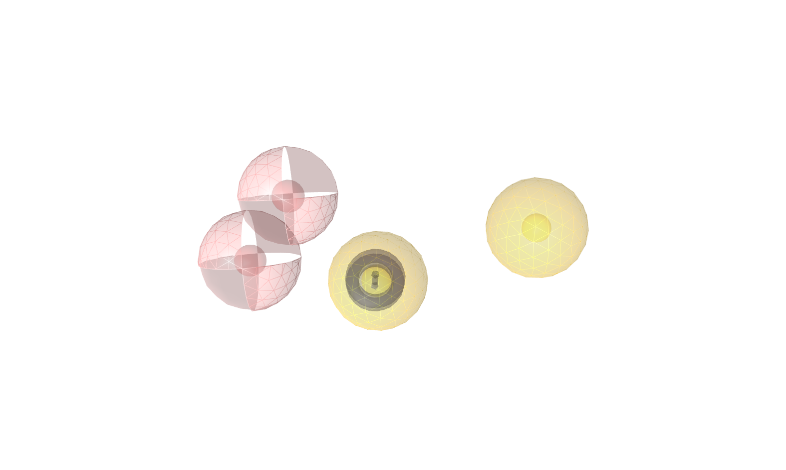** |
| **CTSK** | **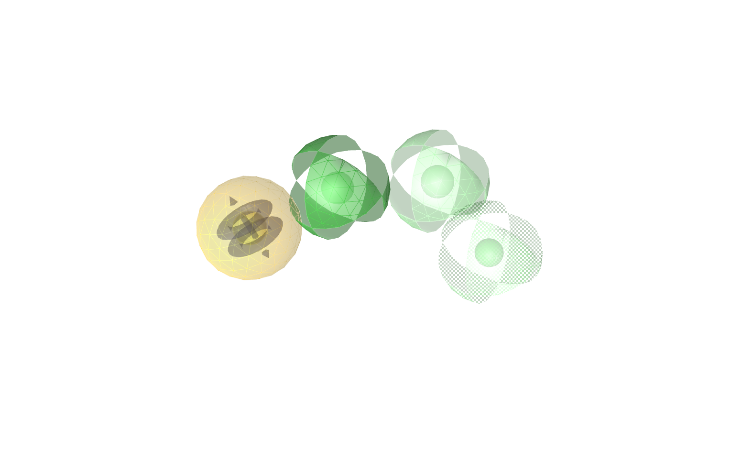** | **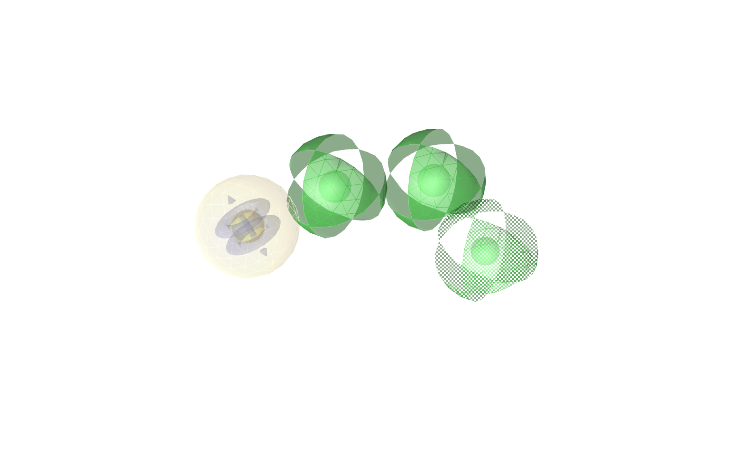** |
| **DHFR** | 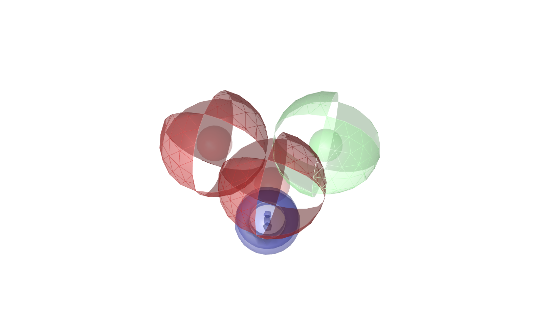 | 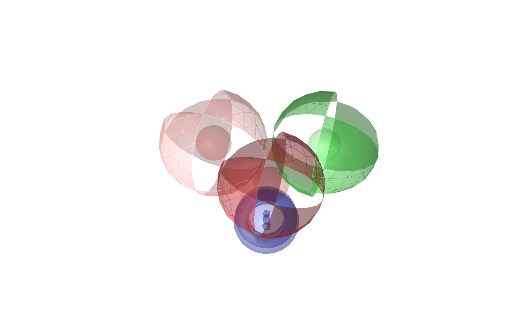 |
| **EGFR** | **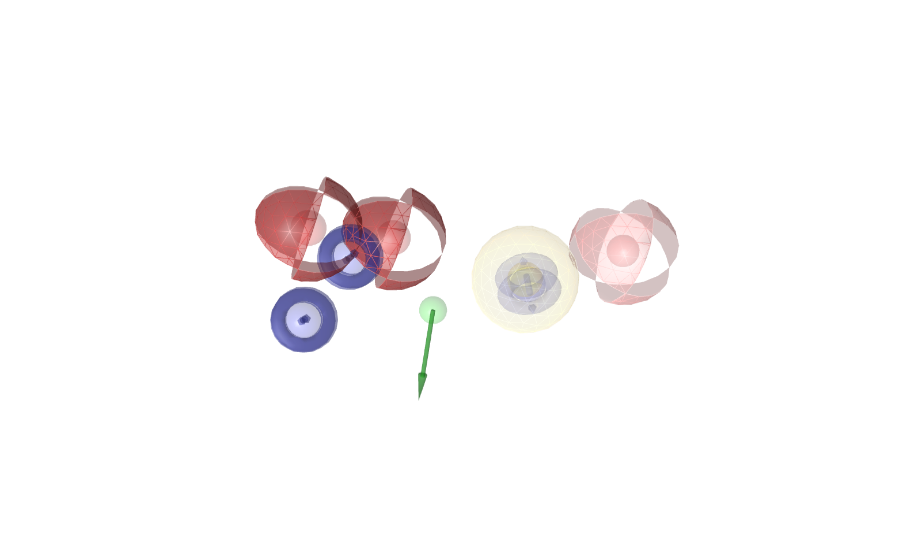** | **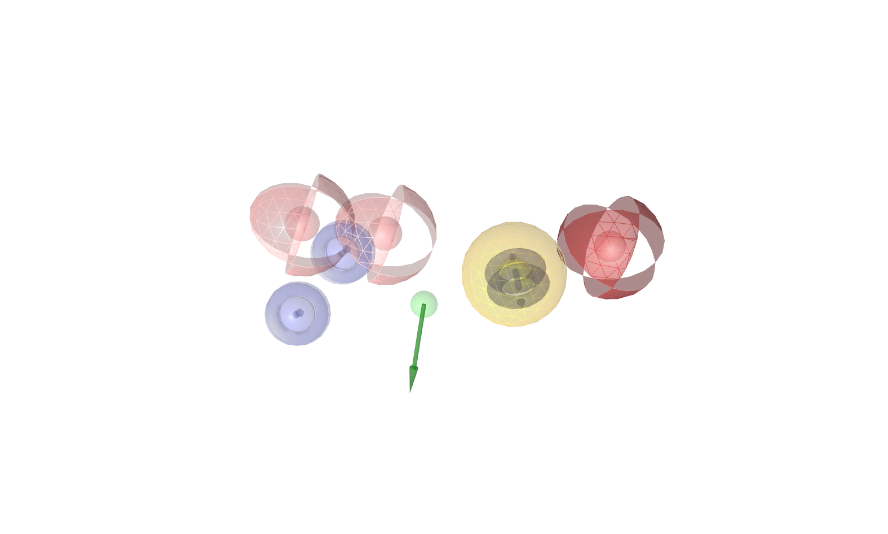** |
| **HMGR** | **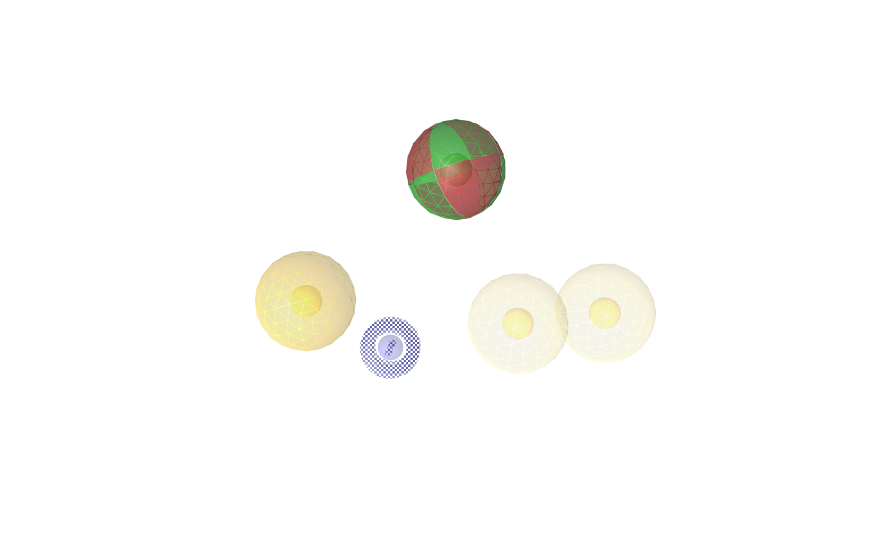** | **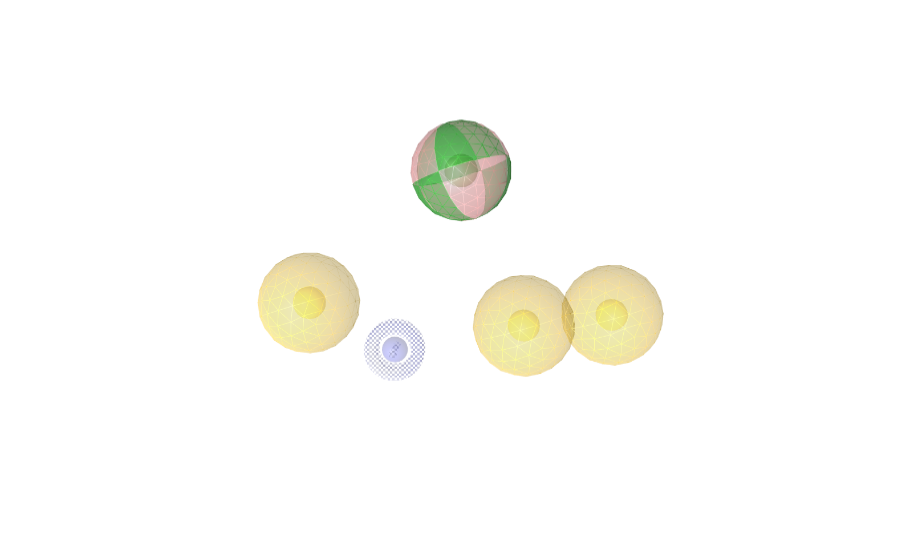** |
| **HSP90** | **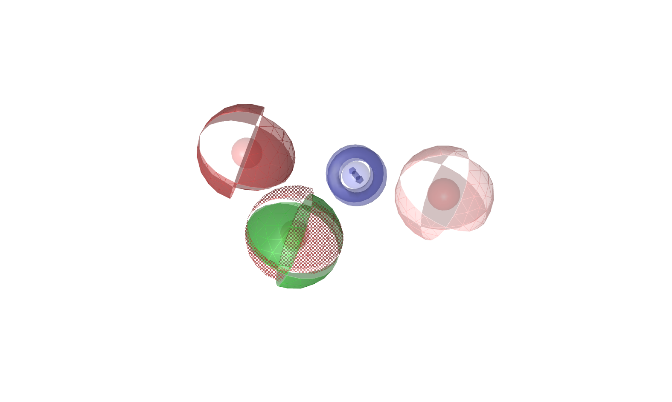** | **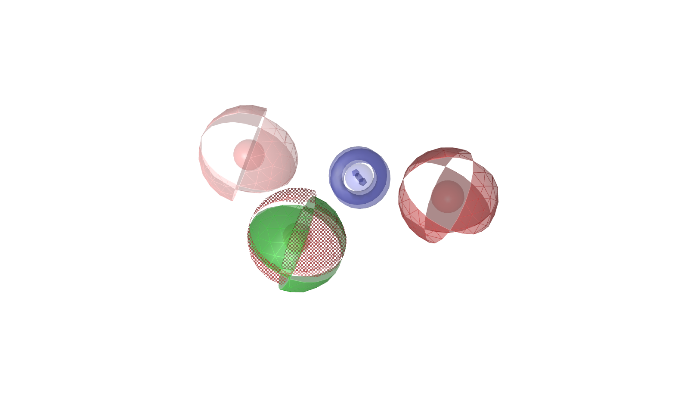** |
| **IGF1R** | **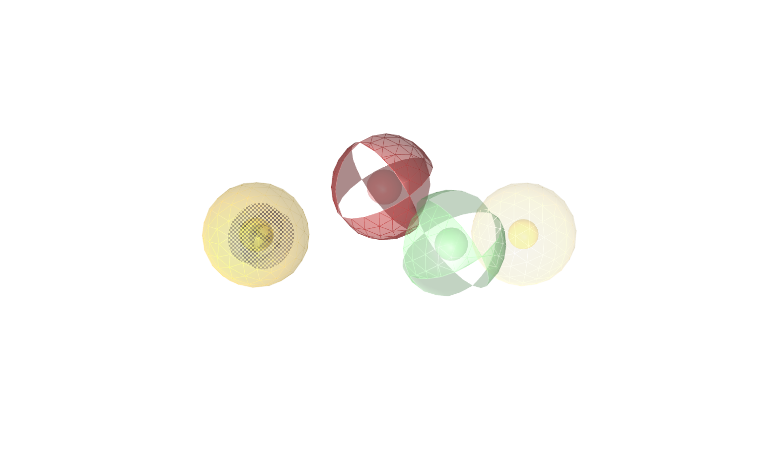** | **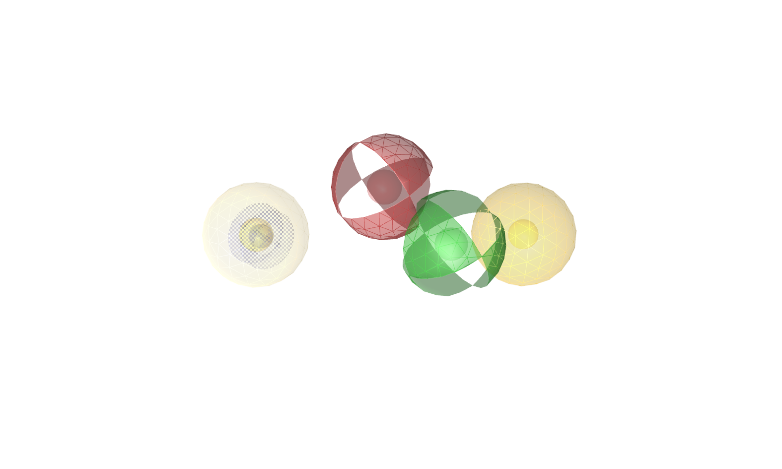** |
| **INHA** | **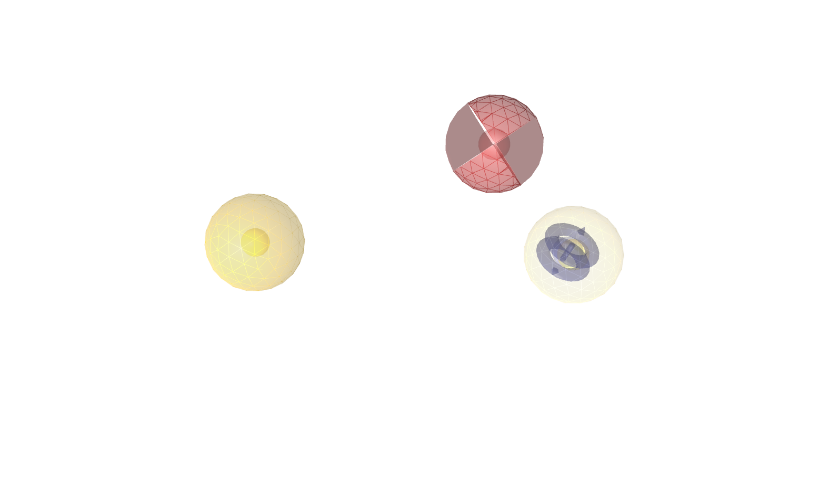** | **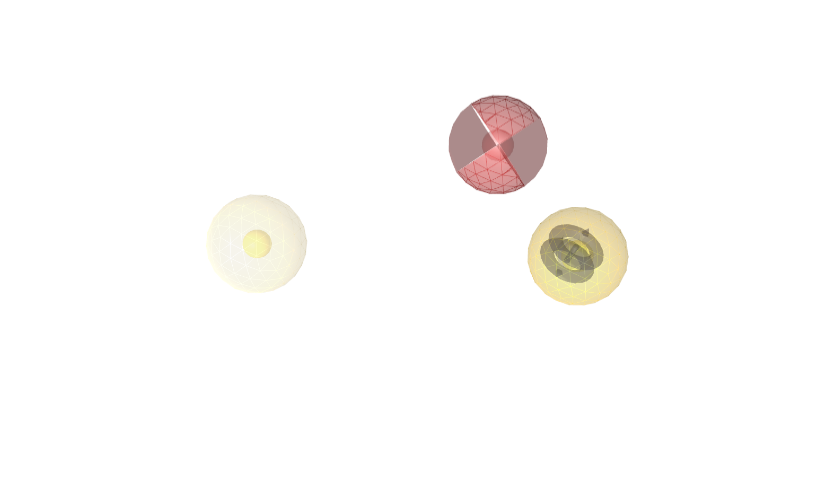** |
| **KIF11** | 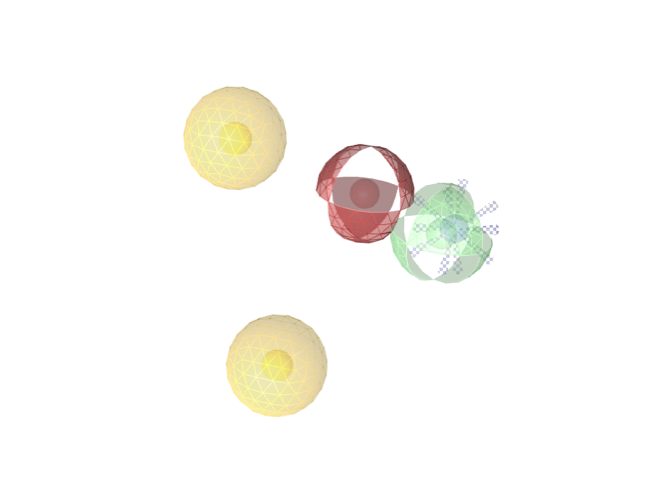 | 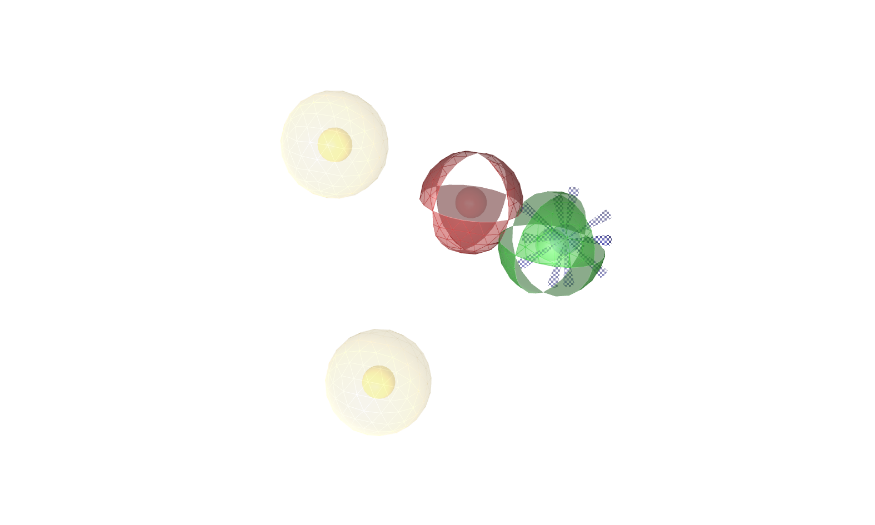 |
| **MDM2** | **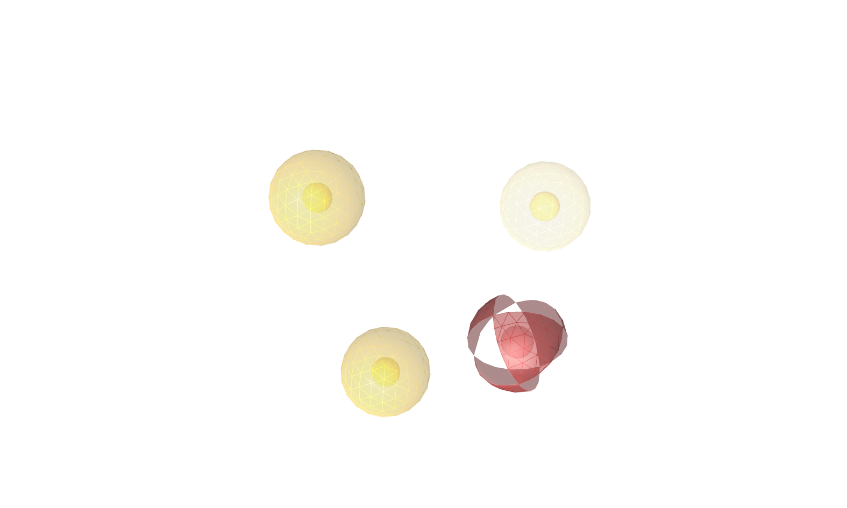** | **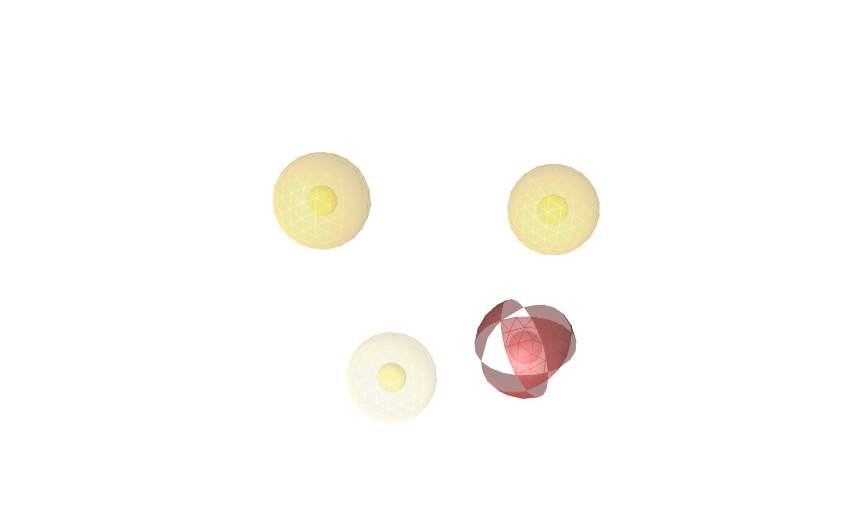** |
| **MK2** | 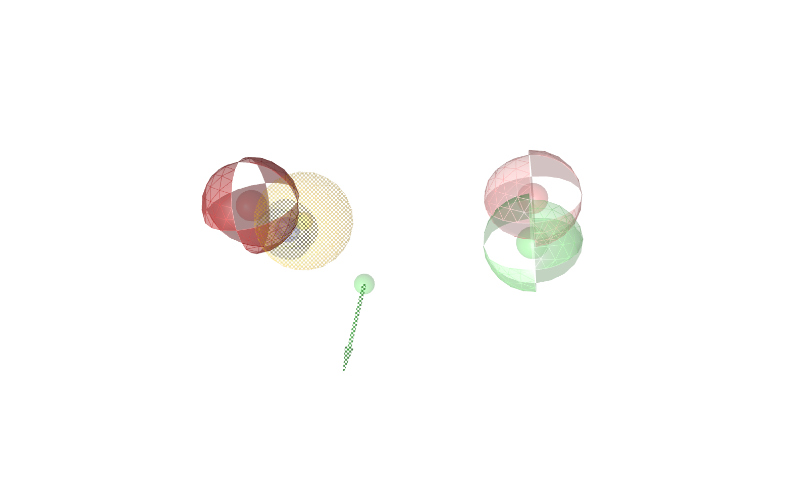 | 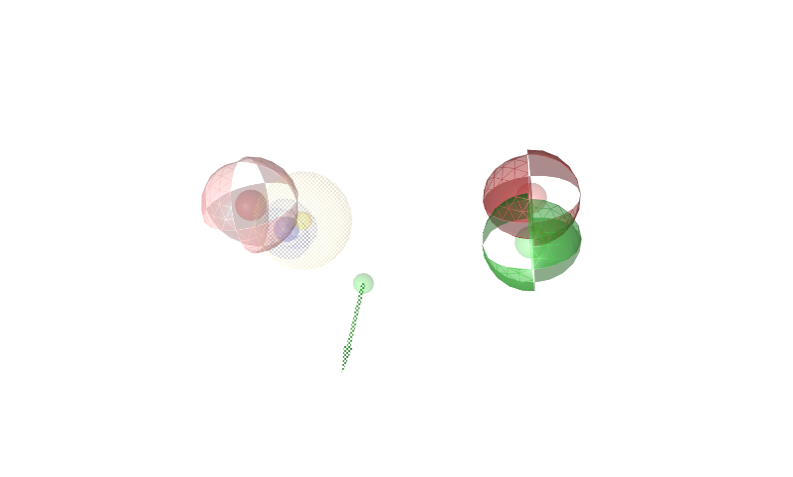 |
| **PIM-2** | 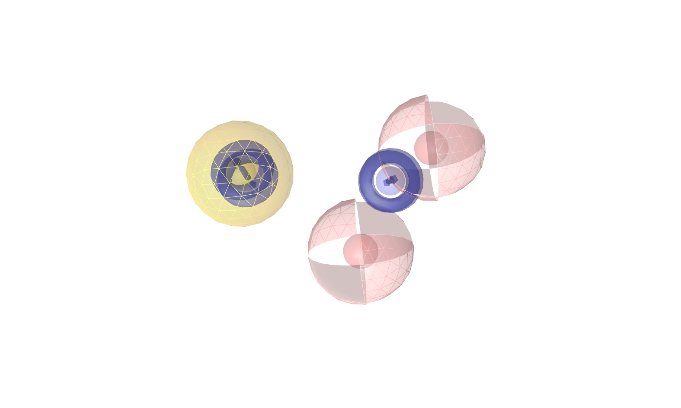 | 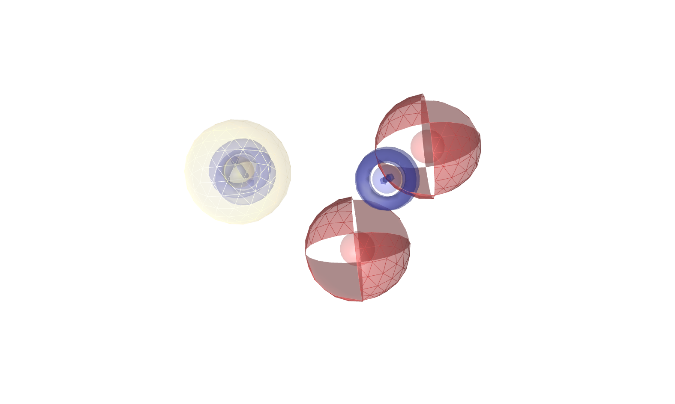 |
| **PRKCQ** | 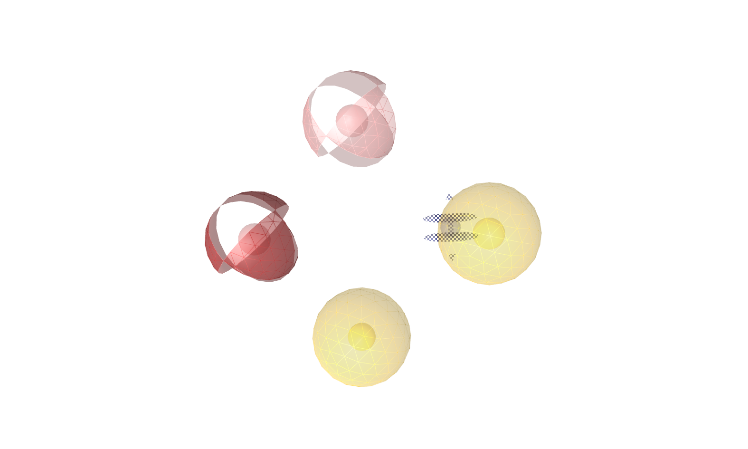 | 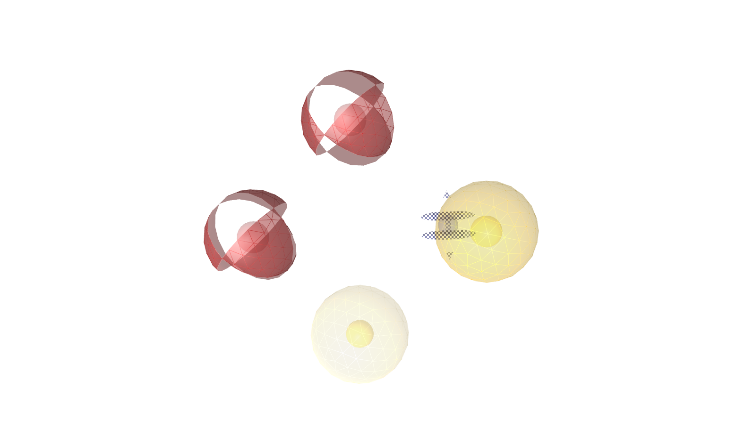 |
| **QPCT** | 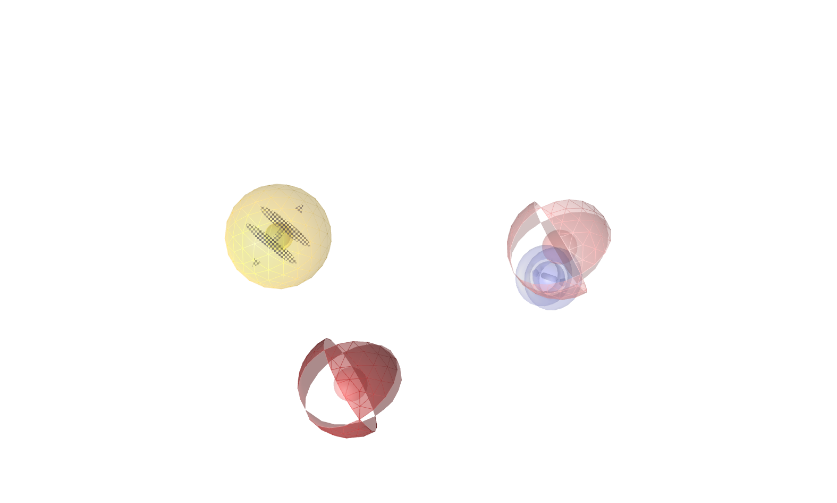 | 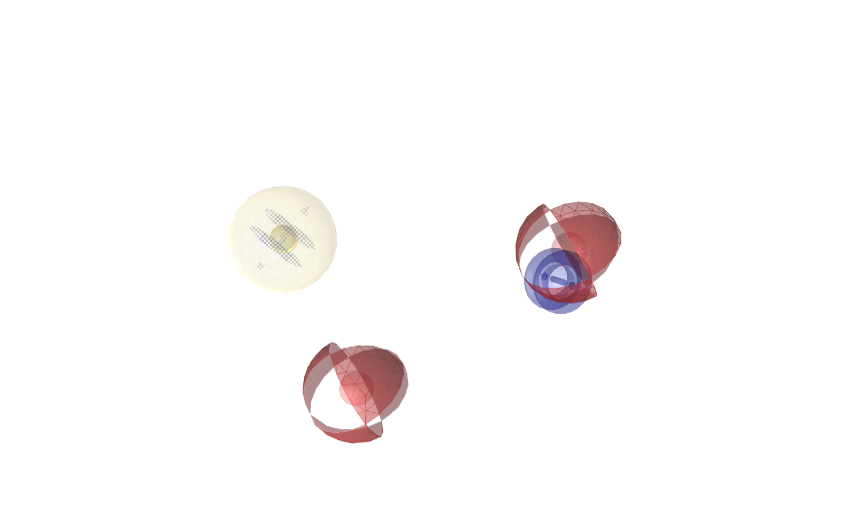 |
| **ROCK-1** | **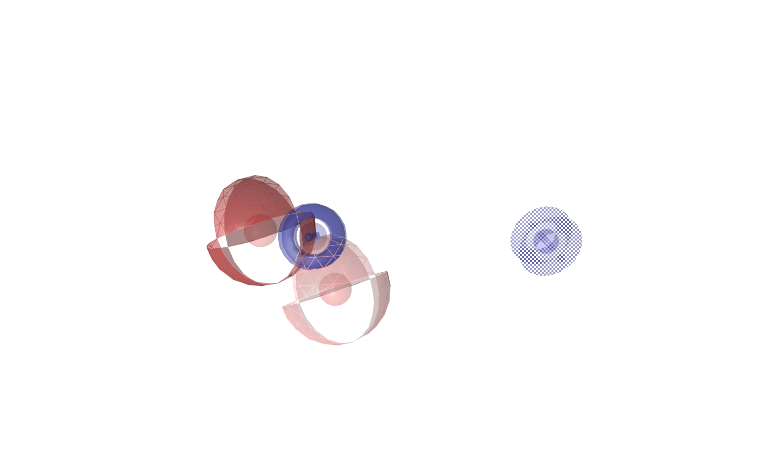** | **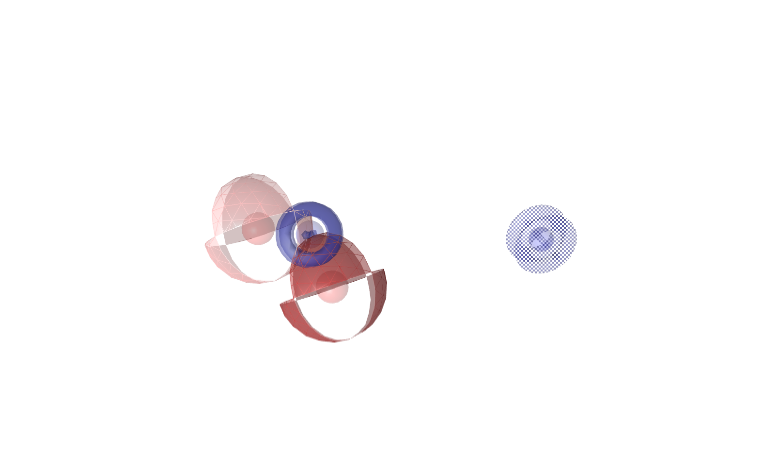** |

Supplementary Figure S1. Set of pharmacophore models included in the VS procedures.

For each of the 20 proteins, a pair of overlapping pharmacophores (described here as *left* and *right*) is shown. The features of each model are colored as follows: hydrogen bond donor - HBD (green), hydrogen bond acceptor - HBD (red), hydrophobic group – HY (gold) and aromatic ring – AR (blue). Features with higher transparency on either the pharmacophore model pairs are disabled to allow the screening of overlapping fragments (NPDFs). Illustration of exclusion volume spheres (XVols) was omitted for clarity.

Supplementary Figure S2. Chemical moieties examples obtained from the VS procedures using pharmacophore models for the proteins CDK2/VEGFR2.

Supplementary Table S1. Molecular descriptors for each compound set before (PRE) and after (POST) the VS protocol. Partition coefficient (clogP), H-bond acceptors (HBA), H-bond donors (HBD), topological polar surface area (TPSA), molecular weight (MW), and molecular complexity were estimated using DataWarrior v5.0.0 (Sander et al., 2015). EXT=extensive fragments; NON=non-extensive fragments; NP=original natural products.

|  |  |  |  |  |  |  |  |
| --- | --- | --- | --- | --- | --- | --- | --- |
|  |  | **cLogP** | **H-Acceptors** | **H-Donors** | **Polar Surface Area** | **Molecular Weight** | **Molecular Complexity** |
| **PRE-EXT** | **range** | **-12.87 - 16.28** | **0 - 19** | **0 - 15** | **0 - 359.9** | **47.06 - 849.29** | **0 - 1.21** |
|  | **average** | **2.9 ± 2.77** | **4.86 ± 2.56** | **2.9 ± 1.95** | **84.69 ± 46.17** | **370.37 ± 159.19** | **0.87 ± 0.15** |
| **POST-EXT** | **range** | **-8.29 - 16.28** | **0 - 18** | **0 - 12** | **0 - 299.36** | **47.06 - 849.29** | **0 - 1.21** |
|  | **average** | **3.14 ± 2.82** | **4.66 ± 2.6** | **2.72 ± 1.97** | **81.00 ± 46.52** | **373.61 ± 169.54** | **0.87 ± 0.15** |
| **PRE-NON** | **range** | **-11.46 - 24.43** | **0 - 22** | **0 - 15** | **0 - 377.42** | **48.07 - 859.25** | **0 - 1.22** |
|  | **average** | **2.68 ± 2.97** | **7.91 ± 3.55** | **3.47 ± 2.38** | **121.92 ± 58.94** | **488.61 ± 160.05** | **0.92 ± 0.14** |
| **POST-NON** | **range** | **-11.46 - 24.43** | **0 - 22** | **0 - 15** | **0 - 377.42** | **48.07 - 859.25** | **0 - 1.22** |
|  | **average** | **2.63 ± 2.97** | **8.00 ± 3.55** | **3.44 ± 2.39** | **122.38 ± 59.25** | **488.84 ± 158.73** | **0.92 ± 0.14** |
| **PRE-NP** | **range** | **-24.04 - 25.69** | **0 - 24** | **0 - 15** | **0 - 459.42** | **56.07 - 891.51** | **0 - 1.25** |
|  | **average** | **2.7 ± 3.28** | **7.26 ± 4.1** | **3.27 ± 2.6** | **116.51 ± 67.92** | **477.47 ± 181.12** | **0.92 ± 0.15** |
| **POST-NP** | **range** | **-22.53 - 25.69** | **0 - 24** | **0 - 15** | **0 - 459.42** | **60.08 - 891.51** | **0 - 1.23** |
|  | **average** | **3.17 ± 3.06** | **7.49 ± 3.99** | **3.59 ± 2.65** | **119.69 ± 65.89** | **492.43 ± 175.63** | **0.92 ± 0.14** |
|  |  |  |  |  |  |  |  |

Supplementary Table S2. Percentage of duplicates with respect to the total number of molecular entities recognized as hit by the original script Fragmenter (S1) or our modification (S2) for the entire set of Left and Right pharmacophore models.

*The percentage values for S2 were estimated by subtracting first the duplicates for S1 and, therefore, excluding any extensive fragment.

|  |  |  |  |  |
| --- | --- | --- | --- | --- |
| **Protein** | **Left Duplicates** | | **Right Duplicates** | |
|  | **S1** | **S2*** | **S1** | **S2*** |
| **ACE** | 10,31 | 5,07 | 8,15 | 6,48 |
| **ACHE** | 14,42 | 1,34 | 11,70 | 5,72 |
| **AURKA** | 10,97 | 3,98 | 12,33 | 11,67 |
| **CATL** | 10,72 | 5,32 | 8,76 | 5,59 |
| **CDK2/VEGFR2** | 5,23 | 8,58 | 5,41 | 5,75 |
| **COX2** | 11,18 | 11,25 | 10,13 | 6,40 |
| **CTSK** | 10,62 | 6,16 | 12,23 | 4,40 |
| **DHFR** | 10,42 | 9,03 | 10,76 | 7,14 |
| **EGFR** | 11,76 | 6,49 | 7,69 | 7,65 |
| **HMGR** | 9,81 | 5,08 | 7,14 | 1,74 |
| **HSP90** | 10,99 | 7,15 | 10,84 | 7,08 |
| **IGF1R** | 9,94 | 4,29 | 10,27 | 4,37 |
| **INHA** | 5,68 | 2,64 | 9,98 | 5,72 |
| **KIF11** | 8,23 | 4,29 | 16,92 | 0,07 |
| **MDM2** | 8,00 | 3,03 | 7,71 | 2,94 |
| **MK2** | 10,39 | 5,35 | 11,48 | 4,40 |
| **PIM-2** | 12,02 | 7,40 | 12,47 | 12,11 |
| **PRKCQ** | 7,59 | 3,89 | 8,85 | 4,55 |
| **QPCT** | 9,84 | 3,49 | 9,23 | 7,73 |
| **ROCK-1** | 6,71 | 2,27 | 9,43 | 4,13 |
|  |  |  |  |  |

Supplementary Table S3. Abstraction layers of pharmacophoric feature constraints. Taken from [441], [442].

| **Layer** | **Classification** | **Universality** | **Specificity** |
| --- | --- | --- | --- |
| 4 | Chemical functionality without geometric constraint, e.g an H-bond acceptor without a projected point or a lipophilic group | +++ | - |
| 3 | Chemical functionality (H-bond acceptor, H-bond donor, positive ionizable, negative ionizable, hydrophobic) with geometric constraint, e.g. an H-bond acceptor vector including an acceptor point as well as a projected donor point; aromatic ring including  a ring plane | ++ | + |
| 2 | Molecular graph descriptor (atom, bond) without geometric constraint, e.g. a geometrically unconstrained phenol group | + | ++ |
| 1 | Molecular graph descriptor (atom, bond) with geometric constraint, e.g. a phenol group facing a parallel benzenoid system within a distance of 2 to 4 angstroms | -- | +++ |
